# Global airborne bacterial community—interactions with Earth’s microbiomes and anthropogenic activities

**DOI:** 10.1073/pnas.2204465119

**Published:** 2022-10-10

**Authors:** Jue Zhao, Ling Jin, Dong Wu, Jia-wen Xie, Jun Li, Xue-wu Fu, Zhi-yuan Cong, Ping-qing Fu, Yang Zhang, Xiao-san Luo, Xin-bin Feng, Gan Zhang, James M. Tiedje, Xiang-dong Li

**Affiliations:** ^a^Department of Civil and Environmental Engineering, The Hong Kong Polytechnic University, Kowloon, Hong Kong;; ^b^Department of Health Technology and Informatics, The Hong Kong Polytechnic University, Kowloon, Hong Kong;; ^c^Shanghai Engineering Research Center of Biotransformation on Organic Solid Waste, School of Ecological and Environmental Science, East China Normal University, Shanghai 200241, China;; ^d^State Key Laboratory of Organic Geochemistry, Guangzhou Institute of Geochemistry, Chinese Academy of Sciences, Guangzhou 510640, China;; ^e^State Key Laboratory of Environmental Geochemistry, Institute of Geochemistry, Chinese Academy of Sciences, Guiyang 550002, China;; ^f^State Key Laboratory of Tibetan Plateau Earth System, Resources and Environment (TPESRE), Institute of Tibetan Plateau Research, Chinese Academy of Sciences, Beijing 100101, China;; ^g^Institute of Surface-Earth System Science, School of Earth System Science, Tianjin University, Tianjin 300072, China;; ^h^College of Resources and Environment, University of Chinese Academy of Sciences, Beijing 100049, China;; ^i^International Center for Ecology, Meteorology, and Environment, School of Applied Meteorology, Nanjing University of Information Science and Technology, Nanjing 210044, China;; ^j^Department of Plant, Soil and Microbial Sciences, Michigan State University, East Lansing, MI 48824;; ^k^Department of Microbiology and Molecular Genetics, Michigan State University, East Lansing, MI 48824

**Keywords:** airborne bacteria, Earth microbiome, bioaerosols, anthropogenic impacts, biogeography

## Abstract

Airborne bacteria are an influential component of the Earth’s microbiomes, but their community structure and biogeographic distribution patterns have yet to be understood. We analyzed the bacterial communities of 370 air particulate samples collected from 63 sites around the world and constructed an airborne bacterial reference catalog with more than 27 million nonredundant 16S ribosomal RNA (rRNA) gene sequences. We present their biogeographic pattern and decipher the interlacing of the microbiome co-occurrence network with surface environments of the Earth. While the total abundance of global airborne bacteria in the troposphere (1.72 × 10^24^ cells) is 1 to 3 orders of magnitude lower than that of other habitats, the number of bacterial taxa (i.e., richness) in the atmosphere (4.71 × 10^8^ to 3.08 × 10^9^) is comparable to that in the hydrosphere, and its maximum occurs in midlatitude regions, as is also observed in other ecosystems. The airborne bacterial community harbors a unique set of dominant taxa (24 species); however, its structure appears to be more easily perturbed, due to the more prominent role of stochastic processes in shaping community assembly. This is corroborated by the major contribution of surface microbiomes to airborne bacteria (averaging 46.3%), while atmospheric conditions such as meteorological factors and air quality also play a role. Particularly in urban areas, human impacts weaken the relative importance of plant sources of airborne bacteria and elevate the occurrence of potential pathogens from anthropogenic sources. These findings serve as a key reference for predicting planetary microbiome responses and the health impacts of inhalable microbiomes with future changes in the environment.

## Significance

Understanding the interactions of planetary microbiomes and their ecological and health consequences requires in-depth knowledge of bacterial communities in the atmosphere, which is the most untouched microbial habitat on the Earth. By establishing a comprehensive atlas of global airborne bacteria, we found that half of the airborne bacteria originate from surrounding environments and are mainly influenced by local meteorological and air quality conditions. One feature of the airborne bacteria in urban areas is that an increasing proportion consists of potential pathogens from human-related sources. The present study defines the aerial microbial world and its origins in a changing climate, and contributes to assessments of the health impact in atmospheric environments.

Airborne bacteria are key components of bioaerosols, which play a vital role in channeling the transmission of microbes across the atmosphere, biosphere, and anthroposphere on the Earth’s surface (1). They are thereby important to the dissemination of microbes, their processes, and to plant and animal health, including humans (2).

Large-scale studies documenting the microbial features in soil (3), ocean (4), and human waste (e.g., wastewater treatment plants) (5) have been systematically conducted. The results show unique microbiomes in each ecological habitat and also suggest an interrelationship between microbiomes in surface environments. However, air has usually been regarded as purely a conduit for terrestrial and aquatic microbial life (6); but it is also a habitat of microorganisms (7), with more than 1 × 10^4^ bacterial cells/m^3^ (2) and hundreds of unique taxa (8). Airborne microbiomes have rarely been documented globally, especially with regard to their community structures, biogeography, anthropogenic impacts, and interactions with Earth’s other microbiomes. A systematic large-scale study can shed light on the central role of the atmosphere in contributing to Earth’s microbial habitats and facilitate predictions of ecosystem responses to environmental changes, for example, climate, air quality, land use, human activities, and so on (9).

The structural distribution of environmental bacteria (3) varies with changes in their environment. For example, microbial diversity in soil is very much influenced by pH and temperature (3, 10), while salinity is a dominant factor in marine systems (4, 11). However, the underlying mechanisms driving the dynamics of airborne bacterial communities have yet to be globally characterized. Hence, research on microbial community structures, biogeographic patterns, and driving mechanisms on a global scale is necessary for understanding atmospheric microbiomes.

Whether in the case of the intraenvironment (atmosphere) or interenvironments (across media), microbes do not live in isolation; rather, they have multiple ecological relationships, ranging from mutualism to competition. Based on theory and ultra-large sample sizes, these interactions have been mathematically modeled as an adjacent matrix (12–14), such as network structures, for soil (15), plant (16), and marine (17) ecosystems, as well as for the human microbiome (18). Yet the important medium of transmission—the air environment—has not been resolved. Moreover, in studies of Earth’s bacterial co-occurrence networks, a gap in understanding remains on the role of airborne bacterial communities in the global microbial world and their interactions with different microbiomes.

Evidence is mounting of anthropogenic impacts on airborne bacterial communities (1, 2, 19), but there is an incomplete global perspective on alterations due to urbanization and the related contributing mechanisms. Yet it is essential to understand these in order to pinpoint the interplay between human activities and natural airborne microbiomes, and to understand the interactions between humans and nature.

To address these knowledge gaps, we acquired and then organized a global airborne bacterial dataset from 76 newly collected air samples (combining 803 weekly samples) and incorporated 294 samples from reputable studies covering 63 worldwide sites. The sampling sites ranged from those on the ground level (1.5 to 2 m high) to rooftops (5 to 25 m high) to high mountains (5,380 m above sea level [a.s.l.]), and from densely populated urban centers to the Arctic Circle, for a more diverse coverage in terms of altitudes and geographic regions than has hitherto been attempted. Our goal was to attain a comprehensive understanding of bacterial biogeographic patterns in macro ecosystems and to assess the degree of commonality and interrelationships among them. We then used data from the Earth Microbiome Project (EMP) (20), involving more than 5,000 samples from 23 varied surface environments, to explore the interconnections between airborne bacteria and other surface-based microbiomes. Considering other factors that could impact airborne bacterial communities, we focused not only on environmental filtering effects, but also on interactions among airborne bacterial communities, external source contributions, and connections with bacteria from other habitats on Earth.

## Results and Discussion

### Structure and Distribution of Global Airborne Bacterial Communities.

#### Structure of global airborne bacterial communities.

**Fig. 1.**
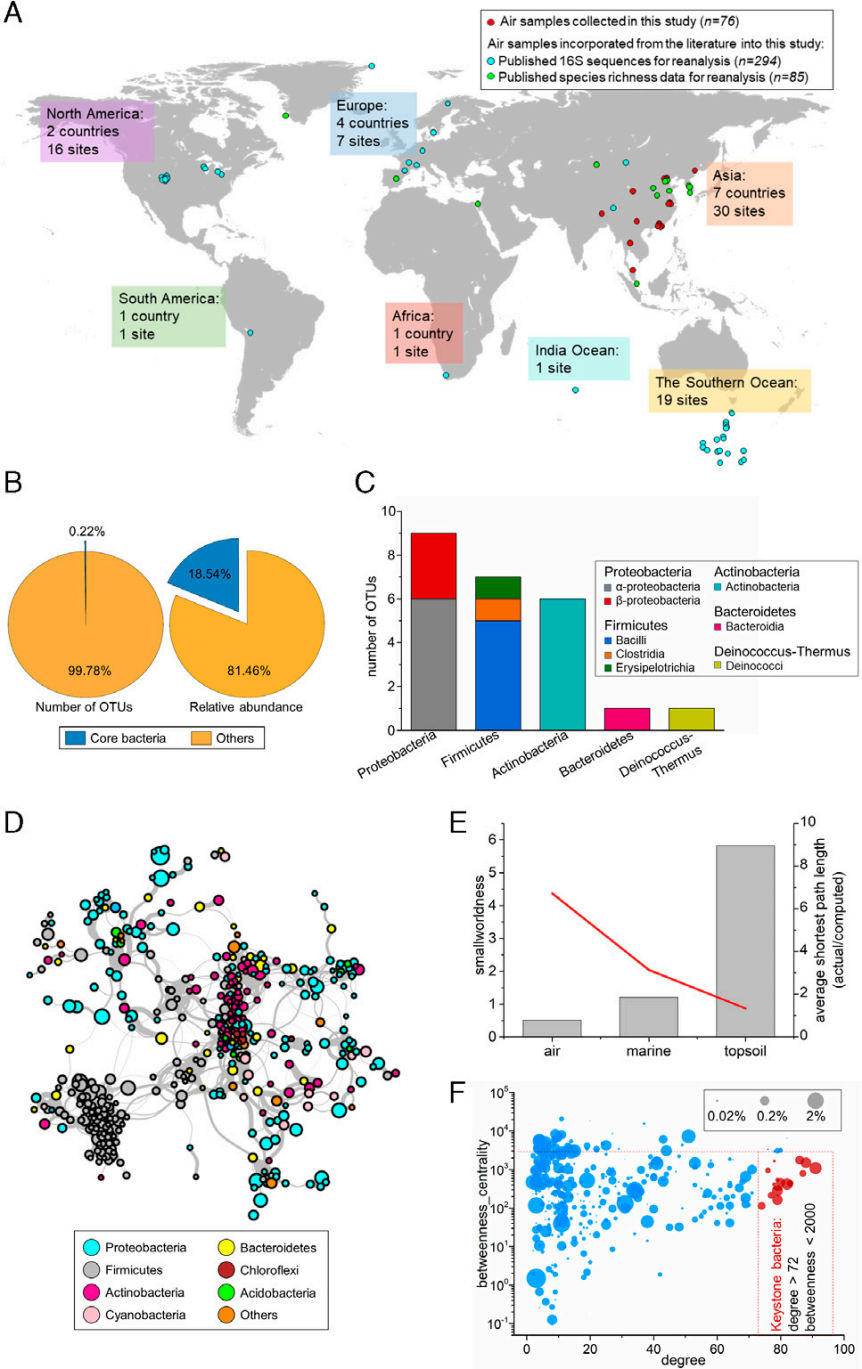
The structure of globally distributed airborne bacterial communities. (*A*) Locations where air samples and environmental data were collected across the globe. (*B*) The number, proportion, and relative abundance of the global core OTUs compared with those of the remaining bacterial OTUs. (*C*) The taxonomic composition of the global core bacteria at the phylum and class level. (*D*) The global airborne bacterial community co-occurrence network. The connections (edges) stand for a strong *(Spearman’s ρ > 0.6)* and significant (*p* < 0.01) correlation. The nodes represent the combined OTUs with unique annotations for genus level in the datasets. The size of each node was proportional to the mean relative abundance across 370 samples. Nodes were colored by the phyla of the bacteria. (*E*) “Small-network” identification based on a “smallworldness” index and the average shortest path length of the global bacterial community network in air, marine, and soil environments. (*F*) Degree—the betweenness centrality plot of each node in the co-occurrence network. The nodes in red are viewed as keystone species. The size of the nodes shows the relative proportions of the OTUs in the total microbiome.

There were 10,897 taxa detected from 370 individual air samples (Fig. 1*A*), and most bacterial sequences belonged to phyla (and subphyla), including Firmicutes (24.8%), Alphaproteobacteria (19.7%), Gammaproteobacteria (18.4%), Actinobacteria (18.1%), and Bacteroidetes (8.6%) (*SI Appendix*, Fig. S1). The abundance–occupancy relationship (AOR) between the number of samples a bacterial taxon occupies and its average abundance within the global air followed a sigmoid curve, which is similar to the widely observed pattern for wild animals and plants. Here, the AOR concept was applied to determine the core subset (abundant and widespread bacteria indicated by both high abundance and high occupancy) in the atmosphere (21) (*SI Appendix*, Fig. S2). The positive AOR revealed a hyperdominant pattern worldwide (22), in which 24 operational taxonomic units (OTUs, an analytical unit grouped by DNA sequence similarity in microbial ecology) (0.22% of the total number of OTUs) accounted for 18.5% of total detected sequences (Fig. 1 *B* and *C* and *SI Appendix*, Table S1). Moreover, we also determined the core communities in marine and topsoil habitats based on the global datasets (3, 4). However, no overlaps were found within the three largest ecosystems, revealing a unique core community in each ecosystem (*SI Appendix*, Table S2).

A global airborne community co-occurrence network was constructed (Fig. 1*D*), encompassing 5,038 significant correlation relationships (Spearman’s ρ > 0.6, *p* < 0.01) among 482 connected OTUs (around 21 edges per node, *SI Appendix*, Table S3). Notably, compared to their counterparts in topsoil and marine environments, airborne bacteria were not closely interconnected, having an average shortest path (intranode connection) length of 5.24. Their clustering approach appeared to be more random. The topology has low resistance to changes (with a “smallworldness” index = 0.51, Fig. 1*E* *and* *SI Appendix*, section S2.1), such as the loss of nodes (bacterial species). Therefore, the observed distant relationships and loose clusters of the network suggest that the airborne bacterial community is more liable to be perturbed as a function of environmental conditions that usually lead to drastic changes in bacterial composition. However, among these loosely interlaced nodes, clustering hub nodes that functioned as the root of a power-law degree distribution network (with more degrees leading to a higher probability of linking) were identified (*SI Appendix*, Fig. S3) (23). Given their significantly higher connection efficiency (Fig. 1*F*) (24), these hub nodes represent keystone species in maintaining the structure of a microbial community relative to their abundances (25). They showed a concentrated distribution (mean correlation coefficient = 0.903). Concretely, each of them was significantly correlated with 15 to 18% of the OTUs in the whole network (*SI Appendix*, Fig. S4 and Table S4 and section S2.2.1). This close-knit community may be a crucial module in the global network, where the keystone species almost dominated the overall topological characteristics (24) (*SI Appendix*, Fig. S4). The functions of keystone taxa in the atmosphere were inferred based on their genetic information or performance in other habitats as summarized in previous studies. Moreover, we found similarities with atmospheric, aquatic, and terrestrial ecosystems among keystone bacterial sets with regard to their compositions and inferred functions (*SI Appendix*, section S2.2.2 and Table S5). This suggests potential associations between airborne bacterial communities and other surface microbial habitats.

#### Biogeographic distribution of global airborne bacteria.

**Fig. 2.**
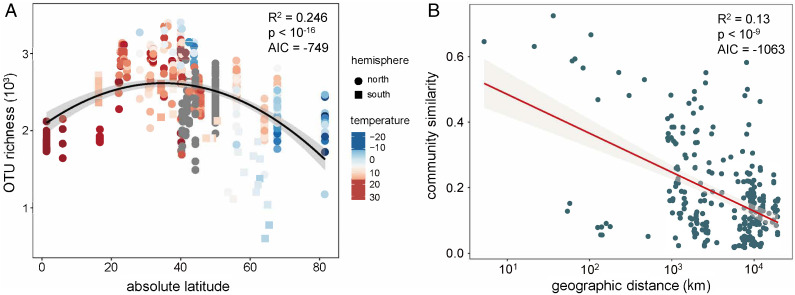
Biogeographic patterns of globally distributed airborne bacterial communities. (*A*) Latitudinal distribution of airborne bacterial diversity (*n* = 455 biologically independent samples). The best polynomial fit was determined on the basis of the corrected Akaike information criterion (AIC) for the given datasets in this study. The line shows the second-order polynomial fit based on ordinary least-squares regression (*R*^2^ = 0.246, *p* < 10^−15^). The color gradient denotes the air temperature corresponding to each sample. Shapes of symbols denote whether a sample originated from the Northern Hemisphere (circle) or the Southern Hemisphere (square). (*B*) Pairwise microbial community similarity (Bray–Curtis) based on relative OTU abundances increases with geographic distance between sampling sites. The red line indicates the least-squares linear regression (*R*^2^ = 0.13, *p* < 10^−9^, AIC = −1,063).

The maximum microbial diversity was observed in the intermediate latitudinal regions (Fig. 2*A*, *R*^2^ = 0.25, *p* < 10^−15^). This was consistent with the two other major types of ecosystems on Earth, that is, soil (3) and water (4), and radically different from the typical latitudinal gradient of diversity (LGD) pattern with macroscopic organisms (26). It has been well documented that the dominant driving factors of latitudinal diversity patterns are pH and soil temperature (3, 10), and the salinity and temperature of water (11). Although quite a few environmental variables affected the number of bacterial taxa (i.e., bacterial richness) in the air (*SI Appendix*, Fig. S5), only air temperature was significantly relevant to latitude (*SI Appendix*, Fig. S6). Temperature could therefore be regarded as the important factor driving the latitudinal diversity distribution (*R*^2^ = 0.064, *p* < 0.005), which is consistent with the role played by air temperature in the diversity reported in a vertical stratification study of airborne microorganisms (11). Hence, we hypothesized that temperature might dominate the uniform parabolic latitudinal diversity patterns of microbial worlds in the three largest ecosystems on Earth (i.e., atmosphere, ocean, and terrestrial systems), and that the source strength may be higher at midlatitudes due to strong winds, erodible surfaces, and heavy pollution (*SI Appendix*, section S2.2.3). Although many regional correlations of total airborne bacterial concentrations with environmental variables were addressed in a previous review (2), most correlations at a global scale were disproven (*SI Appendix*, Fig. S7). In addition, with regard to the similarities among airborne bacterial communities across the globe, local environments generated a distance–decay relationship (DDR) (*R*^2^ = 0.13, *p* < 10^−9^, Fig. 2*B*). Together, our data support the pronounced biogeographic patterns of atmospheric microbiomes, which have also been observed in other ecosystems (3, 5).

### Importance of the Role of Airborne Bacterial Communities in the Microbial World of the Whole Earth.

#### Global airborne bacteria linked with other habitats.

**Fig. 3.**
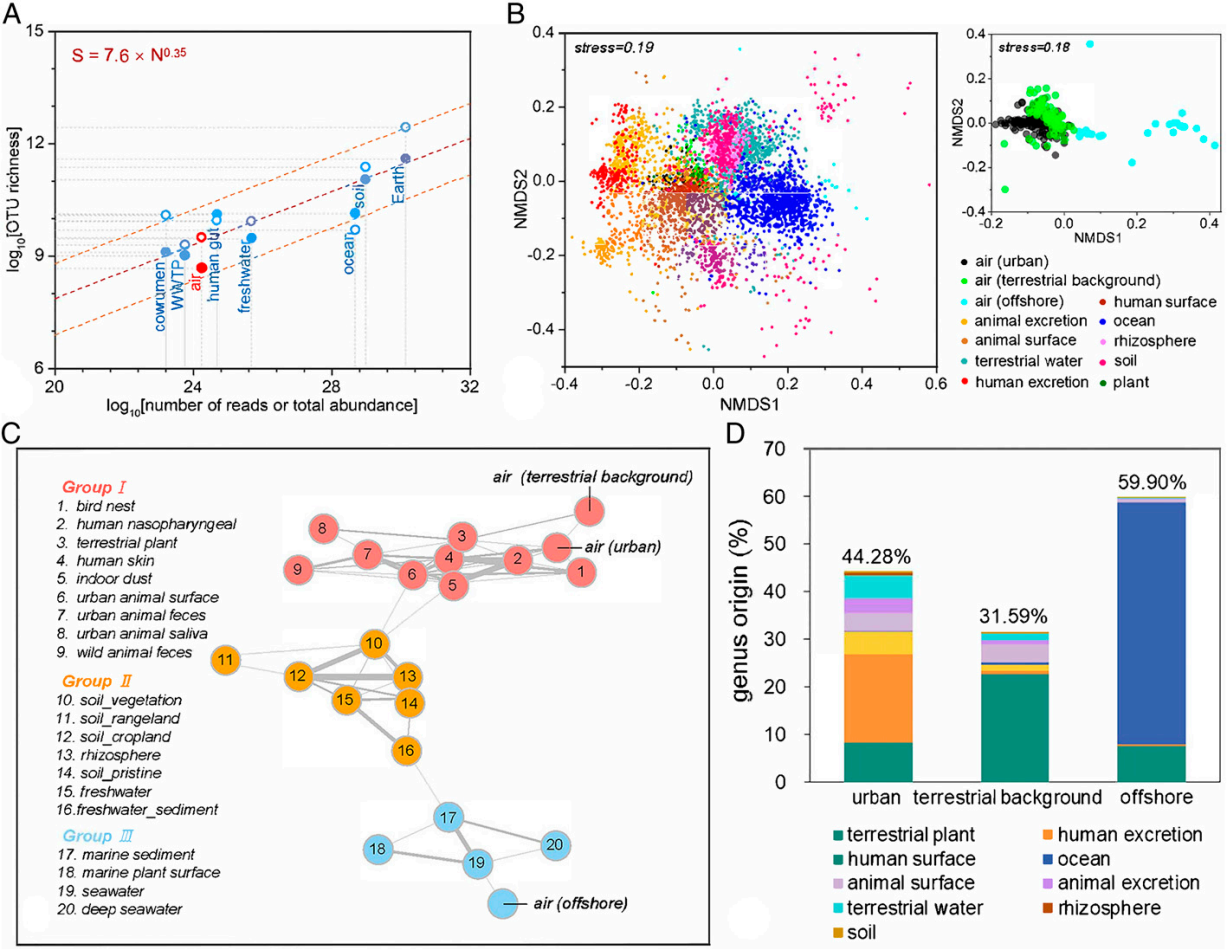
Role of airborne bacteria in the Earth’s microbial world. (*A*) Estimation of the global microbial abundance and richness in various habitats. The global richness (*S*) and the total abundance (*N*) in the corresponding habitats show a scaling relationship (the dashed orange line is the 95% prediction interval). Richness was predicted from the lognormal model using *N*_max_ inferred from our sequencing data (filled circles) or *N*_max_ predicted from the dominance-scaling law (open circles). The estimated *S* and *N* values for each habitat are per se a global sum. Some *S* and *N* were derived from previous studies (5, 28). (*B*) A Bray–Curtis-based nonmetric multidimensional scaling (NMDS) plot showing that different microbial habitats harbor different bacterial communities on the Earth (*n* = 5,189). The Bray–Curtis distance was calculated to represent dissimilarities in the composition of bacterial communities. (*C*) Earth’s bacterial co-occurrence network showing the relationships of interconnection among 23 major microbial habitats. The connections (edges) stand for a strong (Spearman’s ρ > 0.7) and significant (*p* < 0.01) correlation. The thickness of lines represents the value of Spearman’s ρ. The environments were clustered into three groups with different colors by modularization. (*D*) Global airborne bacteria source analysis. Percentage of potential bacterial genera contributions from various environments to airborne bacterial communities in urban, terrestrial background, and offshore areas, respectively, on a global scale.

Estimating the number of species in various global-scale ecosystems can indicate commonness and rarity among taxa, and interconnections across scales of space, time, and abundance (27, 28). The lognormal model was used to predict microbial richness using the total abundances of individuals (*N*) and the quantity of the most dominant taxonomic unit (*N*_max_) according to all known data (28). Although the estimated total abundance of global airborne bacteria (1.72 × 10^24^ cells) was 1 to 3 orders of magnitude lower than that of other habitats, such as soil (9.36 × 10^28^ cells), freshwater (4.70 × 10^25^ cells), and marine (4.68 × 10^28^ cells) habitats, estimates of the bacterial richness of the atmosphere (4.71 × 10^8^ to 3.08 × 10^9^) were comparable to those of the hydrosphere (Fig. 3*A*). As the atmosphere is a less favorable environment for microorganisms than its surface counterparts, the comparably high diversity and complexity of the aerial microbial world presupposes contributions from surrounding habitats, and hence interrelationships with microbiomes in surface ecosystems.

The uniform biogeographic pattern and similar keystone bacterial sets in air, marine, and soil ecosystems suggest interrelationships among bacterial communities in various habitats (Fig. 2*A* and *SI Appendix*, Table S5). Of the 23 major habitats on Earth (5,166 samples from EMP) (20), terrestrial air exhibited more similarity to human- and animal-associated environments, while offshore air bored a closer relationship to oceanic systems (Fig. 3*B*). To further analyze the interactions of airborne bacteria with their counterparts in other habitats, an Earth bacterial co-occurrence network was constructed via the hierarchical agglomeration algorithm (29). As shown in Fig. 3*C*, the 23 habitats were clustered into three groups: human- and animal-associated environments (group I), terrestrial natural environments (group II), and marine environments (group III). This network showed clear gradual transitions and connections: marine–freshwater –soil and rhizosphere–human- and animal-associated habitats. The airborne bacterial communities appeared to be closely associated with their surrounding environments, whose influences were observed to be more pronounced in the settings harboring larger areas of contact with air (Fig. 3*C*), such as seawater (ρ = 0.70, *p* < 0.01), animal surfaces (ρ = 0.72, *p* < 0.01), and human surfaces (nasopharyngeal: ρ = 0.71, *p* < 0.01; skin: ρ = 0.75, *p* < 0.01).

#### Analysis of the sources of global airborne bacteria.

The potential sources of airborne bacterial communities in various regions at the genus level were predicted by SourceTracker2 (30). This program uses Bayesian methods to evaluate all assignments of sink sequences (16S rRNA marker gene sequences in air samples in this case) to all source samples, including an unknown source, and creates a joint distribution of those assignments. Here, the source datasets were retrieved from the Earth Microbiome Project (ftp.microbio.me/emp/) (24). The distribution was sampled to estimate the likelihood that a sequence in an air sample came from a particular source (31). Our results led to a modification of the previous view, based solely on results from the modeling of aerosols in surface ecosystems, that airborne bacteria originated mainly from grasslands, shrubs, and crops (32). Rather, we found that the dominant sources of airborne bacteria were determined by the characteristics of the corresponding surface environment. The major sources at offshore sites were oceanic (56.3 ± 36.3%). Among the onshore sites, human-related sources (23.2 ± 31.5%) contributed greatly to the airborne bacteria in urban areas, dwarfing plant-related sources (22.6 ± 25.2%), which were otherwise dominant in areas of less human impact (Fig. 3*D* and *SI Appendix*, Fig. S8*A*). The large variations in the contributions of human-related sources and terrestrial plants to onshore airborne bacteria were mainly caused by the density of local populations and vegetation coverage, respectively (*SI Appendix*, Fig. S8). Notably, although soil is the most microbiologically abundant (∼10^29^) and diverse (∼10^11^) environment on the Earth (28), its contribution was found to be marginal (<1%), perhaps because of the limited exchange between topsoil and air. The global soil surface area (1.21 × 10^8^ km^2^) (33) was smaller than that of the marine surface (3.62 × 10^8^ km^2^) (34) and leaf surfaces (5.09 × 10^8^ km^2^) (35) which, coupled with the crashing of waves (36) and the shaking of leaves (32), resulted in more exchanges between airborne bacteria and microbiomes in other bacterial habitats than was the case with soil. Although humans and animals may have no advantages in surface areas with air interactions, their frequent activities and constant respiration greatly increase their contact with air, with the result that the dominant source of airborne bacteria is from human- and animal-associated habitats (37), especially in urban areas, a situation that was ignored in previous emission modeling studies (32).

### Anthropogenic Impacts on Global Airborne Bacterial Communities.

#### Human imprints on airborne bacterial communities.

**Fig. 4.**
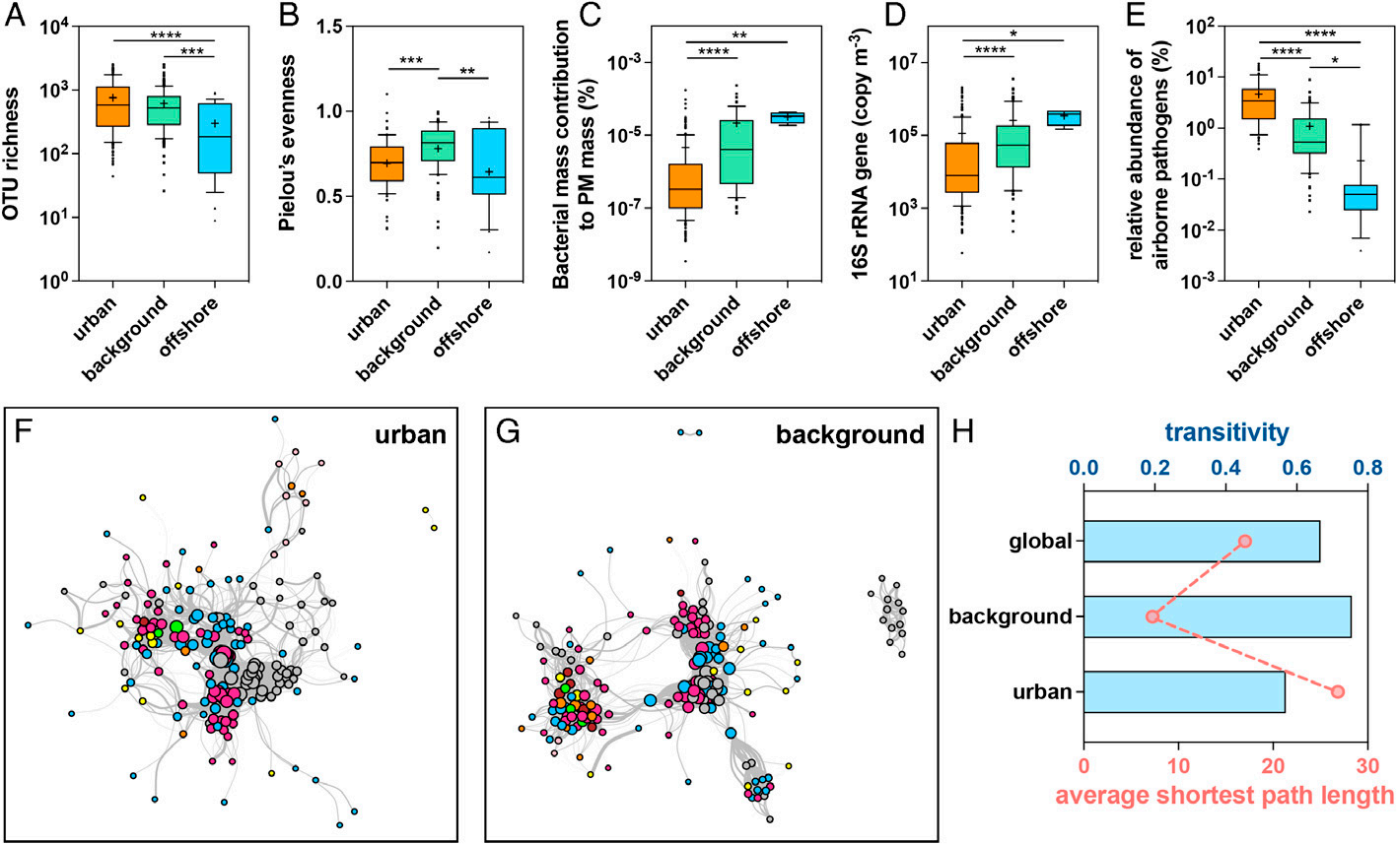
Human imprints on airborne bacterial communities. (*A*–*E*) A comparison of diversity indices (richness and evenness), bacterial mass contributions to PM mass, total airborne bacterial loadings, and the relative abundance of pathogens in urban, terrestrial background, and offshore areas. (*F* and *G*) Co-occurrence networks of airborne bacterial communities in urban and background areas (terrestrial background and offshore areas), respectively. (*H*) Comparisons of network topological characteristics in urban areas, background areas, and the whole global dataset.

The differing structures of airborne bacterial communities between more urbanized and less human-impacted sites are an indication of the significance of anthropogenic influences on airborne bacterial communities (Fig. 3*B*). Nevertheless, no significant disparities in richness between urban and background areas (i.e., areas that are far less impacted by humans, such as our studied sites in remote mountains, offshore environments, and the Arctic region) were detected within the same latitude range (*SI Appendix*, Fig. S9*A*). This suggests that the airborne bacterial richness was mainly controlled by geographic location rather than by anthropogenic impacts. Although humans inhaled a similar number of bacterial species in both urban and natural areas (Fig. 4*A*), the evenness of the bacterial communities was much lower in urban air (Fig. 4*B*), which is reflected in the large increase in abundance of some types of bacteria. For instance, the relative abundance of two typical commensal bacteria, which have some pathogenic species, *Burkholderia* and *Pseudomonas*, was 5.56 and 2.50%, respectively, in urban areas, much higher than in background areas (1.44 and 1.11%). In terms of community composition, urban and background areas both harbored bacteria exclusive to each of those areas (713 and 2,835), although the number of types of bacteria that were found in both areas (4,352) exceeded half of the total number (*SI Appendix*, Fig. S9*B*). Furthermore, the bacterial mass contribution to particulate matter (PM) mass was much lower in urban than in natural areas (Fig. 4*C*), indicating that urbanization increased the proportion of nonbiological particulates, for example, dust and soot, in air PM.

The 16SPIP (16S Pathogenic Identification Process) (38), a comprehensive pipeline designed for clinical samples but also applicable to diverse environmental samples (39, 40), was used to compare potential airborne pathogens in urban and background air. This was used due to the lower sensitivity and accuracy of conventional culture methods based on phenotypes (41). Although the total bacterial loading was lower in urban air (Fig. 4*D*), the relative abundance of potential pathogens was significantly higher (Fig. 4*E*). This was particularly the case with the ESKAPE pathogens (*Enterococcus faecium*, *Staphylococcus aureus*, *Klebsiella pneumoniae*, *Acinetobacter baumannii*, *Pseudomonas aeruginosa,* and *Enterobacter* species) with the highest risk of mortality (42). They exhibited more pronounced abundance in urban air than did other pathogens (*SI Appendix*, Fig. S9*D*). Humans inhaled less abundant airborne bacteria; nevertheless, there is a risk that various pathogenic infections might increase in cities, with 22.4% of identified airborne pathogens (*n* = 37) only having occurred in urban areas (*SI Appendix*, Fig. S9*C*). An additional metagenomic analysis confirmed the composition and abundance of potential pathogens (*SI Appendix*, Fig. S9*D*), although more accurate quantitative diagnostic methods are recommended for future studies, for example, pathogen-specific real-time PCR analysis (43). We hypothesized that the elevated abundance and diversity of airborne pathogens in urban areas might have originated from human-related sources. The alteration of the airborne bacterial taxonomic composition due to urbanization also brought a corresponding change to some phenotypic characters (*SI Appendix*, section S2.3). Moreover, the reduced transitivity and increased average shortest path length in the co-occurrence network of urban airborne bacterial communities indicated that anthropogenic impacts destabilized the network structure (Fig. 4 *F*–*H*).

#### The weakened importance of deterministic processes to microbial community assembly in high-mobility and human-impacted environments.

**Fig. 5.**
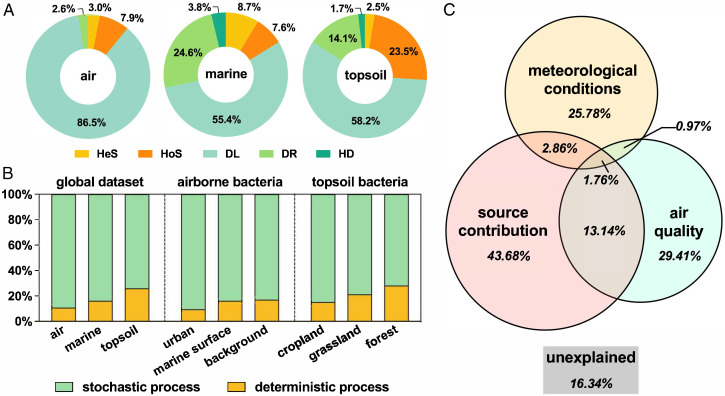
Mechanisms shaping airborne bacterial communities. (*A*) Ecological processes in the assembly of microbial communities inferred by iCAMP. Relative importance of different ecological processes dominating in the assembly of the global airborne (*n* = 370), marine (*n* = 62), and topsoil (*n* = 65) bacterial communities, respectively. DL, dispersal limitation; DR, drift; HD, homogenizing dispersal; HeS, heterogeneous selection; HoS, homogeneous selection. (*B*) Relative importance of deterministic and stochastic processes in different ecosystems. (*C*) VPA showing the relative contributions of air quality, meteorological conditions, and source contributions to variations in the global airborne bacterial communities. The overlap represents the joint effect explained by two or three factor groups together, while the percentage number below each group name represents the variance explained by one group alone. “Unexplained” denotes the variance that could not be explained by any one of these three groups.

Unraveling the ecological drivers controlling community assembly is a central issue. There are two complementary mechanisms of community assembly, namely niche-based deterministic (including environmental filtering, e.g., pH, temperature, moisture, and salinity, and various biological interactions, e.g., competition, facilitation, mutualisms, and predation) and neutral-based stochastic (including birth/death, speciation/extinction, and immigration) (44). To dissect the role of these mechanisms in the airborne community assembly, we employed a recently established quantitative framework (phylogenetic bin-based null model analysis [iCAMP]) (45) to evaluate the relative contributions of each ecological process. This contributed to a further exploration of the mechanisms shaping the structure of microbial communities and biogeographic patterns. As shown in Fig. 5*A*, variations in global bacterial communities were strongly influenced by the dispersal limitation, which exhibited the relative importance of 55.4 to 86.5% in community assembly processes. In this study, the importance of deterministic processes showed a reduced gradient from topsoil (26.0%) to marine (16.2%) and air (10.9%) ecosystems. On the one hand, the wide spread of bioaerosols, coupled with the fact that large particles remain airborne for only a short time, reduced the periods during which bacterial cells were in contact with elements of the environment (e.g., polycyclic aromatic hydrocarbon, heavy metals) and other microbial cells in the air (1). This is a further cause of fewer impacts on airborne bacteria from environmental factors and species interactions in comparison with other habitats, resulting in less significant effects from deterministic processes in shaping airborne bacterial communities. The inconspicuous environmental gradients in the atmosphere due to constant airflow reduced the selection pressure of environmental variables on airborne bacteria (32). Therefore, deterministic processes had less influence on airborne bacterial community assembly than on other ecosystems.

In addition, deterministic processes had less control over the microbial community assembly in human-impacted areas than in natural areas, as seen in atmospheric (urban < offshore ∼ terrestrial background) and topsoil (cropland < grassland < forest) ecosystems (Fig. 5*B*). Frequent human activities can disturb natural environments, reducing the natural environmental gradients (46) and thereby weakening the selections and effects of environmental factors on microbes. Moreover, a destabilized networked microbial structure will lead to an increase in stochastic community assembly, regardless of type of atmosphere (Fig. 4*H*) or habitat (Fig. 1*E*). Consistent with results in the terrestrial atmosphere, coastal airborne microbiomes might be less impacted by environmental filtering and bacterial interactions than ocean areas.

#### Mechanisms shaping airborne bacterial communities.

Through extensive analysis of the direct impacts of 20 different environmental factors on bacterial communities, including diversity, biomass, keystone, core bacterial set, and even the abundance of each OTU (*SI Appendix*, Figs. S7, S10, S11, and S12 and section S2.4), we found that geographic locations, meteorological parameters, and air quality conditions may have influenced the distribution of global airborne bacteria. However, the direct and indirect relationships and causalities among these variables, and the overall contributions of each factor, remain unknown. Structural equation modeling (SEM), which has been widely applied to explore the mechanisms driving microbial communities (3, 5), showed that bacterial communities were affected by multiple factors (*SI Appendix*, Fig. S6*B*). The geographic locations directly impacted airborne bacteria, or indirectly through the effects on some typical environmental factors. The biotic interactions also affected microbial communities, as keystone communities, core communities, and bacterial richness interacted significantly. Finally, we calculated the total effects of environmental filtering (β = 3.06) and bacterial interactions (β = 0.25) on shaping communities. Therefore, in deterministic processes, various biotic and abiotic factors together contributed to the structure and distribution of microbial communities, with the environmental filtering being the main determinant.

Our findings clearly showed that global bacterial communities were strongly driven by stochastic processes, with a relative importance of 89.1, 83.8, and 74.0%, respectively, in atmosphere, ocean, and soil ecosystems (Fig. 5*A  and 5B*). In addition, nearly half of airborne bacteria (averaging 46.3%) were contributed from other environments (Fig. 3*D*), supporting the prominent role of stochastic processes in shaping community assembly (47). Considering not only environmental filtering (deterministic processes) but also source contribution (stochastic processes), we performed a variation partition analysis (VPA) to investigate the integrated mechanisms shaping global airborne bacterial communities (Fig. 5*C*). The analysis showed that airborne bacterial source profiles affected communities most, together explaining 43.7% of the structural variations, a substantially higher percentage than that for air quality (29.4%) and meteorological conditions (25.8%). Due to the extremely dynamic air ecosystem, some key environmental variables suffered from large uncertainties, which also increased the importance of neutral processes in driving airborne bacterial communities (1). The finding that air quality and airborne bacterial source profiles were heavily affected by human activities and explained ∼60% of the variation in community structures corroborated the view that humans impacted airborne bacteria mainly via reduced environmental filtering effects and elevated human-related source contributions. Notably, the three major factor groups significantly affected whole communities, explaining more than 80% of the variations. Thus, the global airborne bacterial communities were mainly impacted by atmospheric environments and the bacterial communities in the surrounding ecosystems.

## Summary

Airborne microbial communities are as complex and dynamic as bacterial assemblages in soil and ocean environments. In our study, the crucial role of airborne bacteria in the Earth’s microbial world was generally ascertained based on their close interactions with bacteria in 23 major surface habitats and the contributions from other ecosystems to nearly half of airborne bacteria. Even though air is a free-flowing ecosystem enabling long-range transport and dynamic processes across geographic barriers, its bacterial community structure appears to be well connected to local environments, especially in terms of the potential source contributions and air quality conditions resulting from human activities. The anthropogenic impacts on airborne bacteria were mainly reflected in fewer biomass loadings, greater potential pathogenic abundance, and more destabilized network structures, arising from the driving mechanisms of reduced environmental filtering effects and elevated human-related source contributions. In summary, this study showed the importance of air in facilitating the exchange of earth microbiomes and provides a theoretical basis for predicting dynamic variations in airborne bacteria in relationship to environmental changes, air pollution, and other human activities at regional or global levels.

## Materials and Methods

### Sample Collection.

We collected 803 air subsamples in Asia mostly on a weekly basis, including those in urban and terrestrial background areas, for an annual cycle (*SI Appendix*, Table S6 for the frequency and numbers per site). Quartz microfiber filters were prebaked for 5 h at 500 °C to remove any contamination caused by carbonaceous material. Most PM_2.5_ samples were collected by high-volume (1,000 L/min) samplers (TH-1000C II, Wuhan Tianhong Instruments) for 24 h, except for the PM_2.5_ samples on Mount Everest, which were collected using an ambient air sampling instrument with a flow rate of 100 L/min for 23.5 h. Total suspended PMs (TSPs) in Thailand and Malaysia were also collected on quartz microfiber filters with a high-volume sampler, and sampling work was performed at a 300 L/min airflow rate for a duration of 24 h. Standard volume was used to calculate the concentration of bacteria in the air. All filter samples were combined into 76 seasonal samples and stored at −20 °C prior to further analysis (no*.* 1–76 in Dataset S1).

### DNA Extraction.

Due to small amounts of DNA, we combined filter samples (field subsamples) belonging to the same season collected in the same site for further analysis. Each filter sample was cut into pieces of roughly 8 × 10 cm for subsequent treatment, and the fragments were extracted with 1× phosphate-buffered saline in 50-mL centrifuge tubes under ultrasonic waves. After sterilizing all of the tools and the 1× phosphate-buffered saline used in the pretreatment process at 120 °C for 20 min, each extract was filtered through a 0.2-μm polyethersulfone (PES) membrane disk filter (47 mm, Pall). The PES membrane disk filter enriched by airborne microbiome was immediately used for the next normal DNA extraction work. The remaining steps were carried out according to the standard FastDNA spin kit for soil (MP Biomedicals) isolation protocol with the exception of the column purification step, which was replaced with magnetic bead purification (Agencourt AMPure XP, Beckman Coulter) for improved yield. All of the steps mentioned above were performed on a clean bench. Once made, the extracted DNA solution samples were stored at −80 °C until further use.

### Library Generation and Sequencing.

The 16S rRNA gene is a widely used marker gene for the classification and identification of bacteria. 16S rRNA amplification, barcoding, pooling, and sequencing library preparation were carried out according to the Illumina protocol (48). The V3 to V4 hypervariable region of the 16S rRNA gene was amplified using KAPA HiFi HotStart ReadyMix (Kapa Biosystems) with degenerate PCR primers, 341F (5′-ACTCCTACGGGAGGCAGCAG-3′), and 806R (5′-GGACTACHVGGGTWTCTAAT-3′) (49). Both forward and reverse primers were tagged with an Illumina adapter, pad, and linker sequences. PCR enrichment was performed in a 50-μL mixture containing a 30-ng template, a fusion PCR primer, and a PCR master mix. Thermal cycling included an initial denaturation at 94 °C for 3 min, followed by 30 cycles of 94 °C for 30 s, annealing at 56 °C for 45 s, and elongation at 72 °C for 45 s, with a final extension for 10 min at 72 °C. The PCR products were purified with AMPure XP beads and eluted in an elution buffer. Libraries were qualified by the Agilent 2100 Bioanalyzer. The validated libraries were used for sequencing on an Illumina MiSeq platform and generating 2 × 300-bp paired-end reads.

### Metadata Collection.

To extend the airborne bacterial communities to a global perspective, we limited our air sample selection to those studies that used a filter-based flow sampler, total DNA extraction, high-throughput sequencing on an Illumina platform, and 16S rRNA gene sequence data. This yielded 294 air samples in the literature (no. 77–370 in Dataset S1) that met our quality criteria were downloaded and processed uniformly. We referred to the quantifications per unit volume of each sample, despite differences in flow rate and sampling time. Altogether, we generated a global airborne bacterial dataset of 370 air samples with different particle sizes (68 PM_2.5_, 171 PM_10_, and 131 TSP) covering 63 sites worldwide including a wide range of latitudes (65.53°S to 81.57°N), altitudes (0 to 5,380 m a.s.l.), climates (15 climatic types following the Köppen−Geiger climate classification system) (47), anthropogenic impacts (e.g., urban, terrestrial background, and offshore areas), and land cover types (*SI Appendix*, Table S7).

We also obtained a global topsoil 16S rRNA gene sequence dataset (3) (*n* = 65, PRJEB19856) and global metagenomic dataset on the surface seawater layer (4) (*n* = 62, PRJEB7988) from the National Center for Biotechnology Information (NCBI) to compare with the airborne microbial communities.

### Sequence Processing.

To minimize the variations associated with sequence processing, all of the collected data on global air, which includes 27,719,673 V3 to V4 hypervariable regions of 16S rRNA gene amplicon reads from 370 combined air samples, were processed uniformly as previously described using mothur (v1.42) (50). Briefly, any chimeric sequences were removed using the VSEARCH tool based on the UCHIME algorithm for quality control (51). Sequences were then split into OTUs at the 97% similarity threshold using the UPARSE pipeline. OTUs were taxonomically annotated with an 80% confidence cutoff, using SILVA (v123) as the reference database (52). To assess potential bacterial pathogens mapped at the species level, raw sequences for each sample were also processed against pathogenic sequences through the 16SPIP pipeline with a criterion of greater than 99% similarity (38). The reliability of identifying pathogens based on paired reads of the V3 to V4 region of the 16S gene has been validated by using Beijing hospital samples identified by a combination of culture and whole-genome shotgun metagenomic analyses (38). Phenotypic information was generated through a multivariate data analysis from METAGENassist (53). The global topsoil 16S rRNA gene sequences were also reanalyzed following the above procedure.

### Other Methods.

Details of other methods used in this study are described in the *SI Appendix*, *SI Materials and Methods*, including 1) the acquisition of environmental data, 2) quantification of target genes, 3) pathogen identification based on metagenome, 4) chemical analysis, and 5) various statistical analyses.

## Supplementary Material

Supplementary File

Supplementary File

## Data Availability

DNA sequence data have been deposited in Sequence Read Archive of the NCBI (https://www.ncbi.nlm.nih.gov/bioproject/PRJNA757592) (54). All other study data are included in the article and/or *SI Appendix*.
