# Supplementary material for: Global airborne bacterial community—interactions with Earth’s microbiomes and anthropogenic activities
Source: Proc Natl Acad Sci U S A. 2022 Oct 10;119(42):e2204465119. doi: 10.1073/pnas.2204465119 (PMC9586312; doi:10.1073/pnas.2204465119)
Supplement: Supplementary File [file pnas.2204465119.sapp.pdf]

PNAS
www.pnas.org

Supplementary Information for

Global airborne bacterial community–interactions with Earth's microbiomes and anthropogenic activities.

Jue Zhao, Ling Jin, Dong Wu, Jia-wen Xie, Jun Li, Xue-wu Fu, Zhi-yuan Cong, Ping-qing Fu, Yang Zhang, Xiao-san Luo, Xin-bin Feng, Gan Zhang, James M. Tiedje*, and Xiang-dong Li*

James M. Tiedje*
Email: tiedjej@msu.edu
Xiang-dong Li*
Email: cexdli@polyu.edu.hk

This PDF file includes:

Supplementary materials and methods
Supplementary results
Figures S1 to S19
Tables S1 to S7
SI References
Additional datasets 1 (SI – B, a separate file)

Table of contents

Supplementary Information Text

S1. SUPPLEMENTARY MATERIALS AND METHODS S5
S1.1 Acquisition of Environmental Data S5
S1.2 Real-Time qPCR Quantification of Targeted Genes S5
S1.3 Pathogen Identification Based on Metagenome S5
S1.4 Chemical Analysis S6
S1.5 Statistical Analyses. S6
S1.5.1 Core Bacteria Identification S6
S1.5.2 Diversity Analyses and Correlations with Environments S6
S1.5.3 Network Construction, Topological Property Calculation, and Keystone Species Identification S6
S1.5.4 Estimation of the Richness of Global Microbiomes S7
S1.5.5 Interactions of the Bacterial Community Composition of Air with Other Bacterial Habitats S9
S1.5.6 Quantifying the Relative Importance of the Microbial Community Assembly Process S9
S1.5.7 Multivariate Analysis S9
S2. SUPPLEMENTARY RESULTS S11
S2.1 Small-world Characteristics in the Global Air, Soil, and Marine Bacterial Co-occurrence Network. S11
S2.2 Composition and Structure of Airborne Bacterial Communities S11
S2.2.1 Differentia of Taxonomic Composition in Whole, Core, and Keystone Bacterial Communities on a Global Scale S11
S2.2.2 Keystone Taxa Associated with Evolutional and Ecological Functions S11
S2.2.3 Airborne Bacterial Diversity Pattern in Mid-latitudinal Regions S12
S2.3 Impacts of Urbanization on Airborne Bacterial Genotypes S13
S2.4 Environmental Impacts on Global Airborne Bacterial Communities S13
S2.4.1 Relationships between Environmental Factors and Airborne Bacterial Diversity and Biomass S13
S2.4.2 Environmental Filtering Impacting Each OTU in Whole Global Airborne Bacterial Communities S13
S2.4.3 Impacts of Environmental Variables on Keystone and Core Bacterial Communities S14

Supplementary Figures

Fig. S1. Airborne bacterial abundance distributions across the globe S16
Fig. S2. Abundance-occupancy relationship S17

Fig. S3. Comparison of networked bacterial communities in the atmosphere, global topsoil, and top marine layer....S18

Fig. S4. Role and composition of the global-scale core and keystone bacterial communities in the atmosphere....S19

Fig. S5. Relationships of the global airborne bacterial diversity index (OTU richness) with air quality and meteorological conditions....S20

Fig. S6. Impacts of environmental filtering on airborne bacterial communities....S21

Fig. S7. Relationships of global airborne bacterial biomass (16S rRNA gene copy number) with geographic locations, air quality, and meteorological conditions....S22

Fig. S8. Global airborne bacterial source contribution....S23

Fig. S9. Anthropogenic impacts on the composition of airborne bacterial and pathogenic communities....S24

Fig. S10. Effect of particulate matter size on airborne bacterial richness and total biomass....S25

Fig. S11. Map showing the geographical locations of the sampling sites for estimating the mean airborne bacterial density.....S26

Fig. S12. RDA identifying the relationships between core & keystone bacteria and soluble ions & heavy metals in air samples....S27

Fig. S13. Airborne bacterial richness (y-axis) and PM concentration (x-axis) plot of samples collected in the mid-latitudinal regions....S28

Fig. S14. Structure of global airborne bacterial cells in urban, terrestrial background, and offshore areas....S29

Fig. S15. Adaptation of global airborne bacteria to local environments in urban, terrestrial background, and offshore areas....S30

Fig. S16. Gram types of global airborne bacteria in urban, terrestrial background, and offshore areas....S31

Fig. S17. Motility of global airborne bacteria in urban, terrestrial background, and offshore areas....S32

Fig. S18. Correlation distribution between airborne bacterial community composition and environmental factors (air pollutants and meteorological conditions)....S33

Fig. S19. Impacts of environmental factors on core and keystone bacterial communities in the global atmosphere....S34

Supplementary Tables

Table S1. The taxonomic composition of 24 global airborne core bacteria (phylum, class, order, family, and genus level)....S35

Table S2. Taxonomic information and relative abundance of core communities in air, marine, and soil habitats based on the global datasets....S36

Table S3. The topological properties of the co-occurrence network....S37

Table S4. The taxonomic composition of 19 global airborne keystone bacteria (phylum, class, order, family, and genus level) S38

Table S5. Summary of keystone taxa reported in the literature on different ecosystems and habitats S39

Table S6. Sampling frequency and number of air subsamples in this study S40

Table S7. The overview of climate types and land cover types of 370 air samples in 63 sampling sites S41

Supplementary references..........S44

S1. SUPPLEMENTARY MATERIALS AND METHODS

S1.1 Acquisition of Environmental Data

In this study, all environmental variables were divided into three major groups: meteorological condition, air quality, and earth surface type. The global hourly meteorological data in each site during the sampling process, containing air temperature, air pressure, relative humidity, wind speed, and wind direction; and the corresponding air quality data including AQI, PM_{10}, $PM_{2.5}$, SO_2, NO_2, O_3, and CO, were obtained from diverse national public websites and the environmental monitoring centers of local governments. The global hourly meteorological data in each site during the sampling process were downloaded from the official website of the National Climatic Data Center (ftp://ftp.ncdc.noaa.gov/pub/data/noaa/isd-lite/) and Weather Underground (https://www.wunderground.com/). Since there is still no globally uniform air quality monitoring system like that of NOAA (the National Oceanic and Atmospheric Administration) for air pollutant records, we compiled air quality data obtained from the environmental monitoring centers of the respective national governments, including the United States Environmental Protection Agency (EPA: https://www.epa.gov/), China National Environmental Monitoring Centre (CNEMC: http://www.cnemc.cn/), European Environmental Agency (EEA: https://www.eea.europa.eu/), the Royal Thai Pollution Control Department (https://www.ccacoalition.org/en), the South Africa Air Quality Information System (http://saaqis.environment.gov.za/), the Malaysian Department of the Environment (DOE: https://www.doe.gov.my/portalv1/en/), the Government of Peru (Gob.pe: https://www.gob.pe/), and Environment and Climate Change Canada (http://data.ec.gc.ca/data/).

The quantification of types of land cover in a diameter range of the sampling sites (50 km) was performed with a MODIS (Moderate-resolution imaging spectroradiometer) land cover approach (5′ × 5′ resolution). Using the IGBP (International Geosphere–Biosphere Programme) system (MCD12Q1-1), the different MODIS land coverages were described (1). The relative contribution of each type of landscape to the aerial emission of bacterial cells was predicted by weighting these relative surfaces by their associated bacterial cell concentration, as reported earlier (2).

S1.2 Real-Time qPCR Quantification of Targeted Genes

The total bacterial loading was approximated by the concentration of 16S rRNA gene copies in the air. The 16S rRNA gene was amplified on a StepOnePlus Real-Time qPCR System (Applied Biosystems) with the following primer sequences: 5’-TCCTACGGGAGGCAGCAGT-3’ as the forward primer and 5’-GGACTACCAGGGTATCTAATCCTGTT-3’ as the reverse primer. To quantify the absolute number of copies of 16S rRNA genes in DNA samples, a seven-point standard curve (including a blank standard) in a 10-fold serial dilution was run with samples. All of the samples, standards, and blanks were run in triplicate with a 90% - 110% application efficiency. Finally, the specificity of the amplicons was verified by a melt curve analysis. The 20-μL qPCR reaction mixture contained 10 μL of Power SYBRTM 5 Green PCR Master Mix (Life Technologies, CA, USA), 1 μL of template DNA, 0.5 μL of each primer (100 nM), and RNAse-free water to complete the final 20 μL volume. The 16S rRNA gene was amplified according to the following protocol: an initial step at 95 °C for 10 min for enzyme activation, then 40 cycles of 10 s at 95°C, and 1 min at 60°C for hybridizations and elongations. The amplicon length was around 400-500 bp. In addition, to reduce the variations in the 16S rRNA gene copies caused by particle size, the mean ratios of bacterial loadings in $PM_{2.5}$ with PM_{10} (1: 1.56±0.74) and with TSP (1: 7.44±3.86) collected in the same sites during the same sampling period were used to modify the data drawn from the literature (Fig. S10).

S1.3 Pathogen Identification Based on Metagenomes

The limited DNA content (~50 ng) in each collected ambient air sample was used to construct a low-input library, followed by shotgun sequencing on an Illumina Hiseq X Ten platform with a 150-bp paired-end read length. The clean data were uploaded to the NCBI with the accession number PRJNA858396. The raw reads on metagenomic sequencing were processed to filter out low-quality

reads using fastp with default parameters (3). The filtered clean metagenomic reads were taxonomically profiled using Kraken 2 (v2.0.8-beta) (4) and Bracken (v2.5.0) (5), using the standard Kraken 2 database. Human pathogens, especially the nosocomial ESKAPE pathogens that exhibit multidrug resistance and virulence (*Enterococcus faecium*, *Staphylococcus aureus*, *Klebsiella pneumoniae*, *Acinetobacter baumannii*, *Pseudomonas aeruginosa*, and *Enterobacter* spp.), were identified on the species level to verify the results of the identification of pathogens in the 16SPIP pipeline.

S1.4 Chemical Analysis

After acid pretreatment for filter samples, the final solutions were analyzed by inductively coupled plasma - optical emission spectrometry (ICP-OES) to detect the total concentrations of various elements. Organic carbon (OC) and element carbon (EC) concentrations were analyzed by the DRI Thermal/Optical Carbon Analyzer (Model 2001) and calculated based on the result of thermal optical reflectance (TOR) protocol (6). Soluble anions (NO_3^- and SO_4^{2-}) and cations (NH_4^+) in filtrates were analyzed by Ion Chromatography.

S1.5 Statistical Analyses

S1.5.1 Core Bacteria Identification. The determination of a global core set of airborne bacteria was based on their abundance and occupancy in regional and temporal variations with reference to multiple reported methods. First, 166 OTUs with a high mean relative abundance (> 0.01%) across 370 samples were selected as overall abundant OTUs (7). Then, 68 OTUs among the overall abundant OTUs with an occurrence frequency in all samples of more than 80% were filtered out as widespread OTUs (8). Finally, the dominant OTUs in more than half of the samples were further filtered out and identified as the final core airborne bacterial community. In sum, the process was as follows: OTUs were sorted based on their abundance in each sample, and the accumulated sum of the relative abundance was calculated one by one; those OTUs that made up the top 80% of the bacterial community in each sample, as well as those that occurred in more than half of the samples, were chosen as the final core bacteria.

S1.5.2 Diversity Analyses and Correlations with Environments. To compare the structure of the bacterial community across different regions, α-diversity and β-diversity were computed using the "Picante" package (9) in R based on the original OTU table directly generated by sequence processing. In addition, to minimize deviations in the number of bacterial taxa (*i.e.,* richness) caused by different particle sizes, we used the mean ratio of richness in $PM_{2.5}$ to PM_{10} (1: 1.73 ±0.63) and TSP (1: 2.14±0.84) collected in the same sites during the same sampling period to modify the richness data in the literature (Fig. S10). The Bray–Curtis dissimilarity matrix for the airborne bacterial community structure (OTU abundance-based) between pairs of samples was calculated to estimate the taxonomic β-diversity using the "vegdis" function in the "vegan" R package (10).

Another R package, "geosphere", was applied to calculate the geographic distance between any two sampling sites across the globe, based on geographic coordinates (11). For each environmental variable, a partial Mantel test with 999 permutations was performed to examine the correlation (Pearson's rank correlation) between the environmental variable matrix and the composition of the bacterial community using the "vegan" R package (10).

Also, we used the Akaike information criterion (AIC) to select the best model to fit the relationship between two variables. Given a collection of models for the data, AIC can be used to estimate the quality of each model, relative to each of the other models. AIC has been widely used for statistical inference and is gradually forming the basis of a paradigm for the foundations of statistics.

S1.5.3 Network Construction, Topological Property Calculation, and Keystone Species Identification. To filter out rare OTUs, those OTUs with a mean relative abundance of over 0.1% in all samples and a relative abundance of over 0.5% in any one sample were retained for network

construction. The co-occurrence patterns were based on the Spearman correlation matrix performed with the WGCNA package (12). The final network was constructed with statistically significant relationships ($p < 0.01$, $R > 0.6$) (13). The nodes in this network represent OTUs, whereas the edges (that is, connections) correspond to robust and significant associations between OTUs.

Topological properties were respectively calculated for each node in the resulting network with the "igraph" R package (14), and those OTUs with high degree (> 72, 75% of the highest degree) and low betweenness centrality scores (< 2000, 10% of the highest betweenness centrality) were recognized as the keystone taxa (15). Included in the set of topological properties for each node were degree, betweenness centrality, closeness centrality, and transitivity. Betweenness centrality reveals the role of a node as a bridge between components of a network, while degree reveals the role of a node with direct connections with other OTUs in the whole community. Thus, the two important indexes were normally selected as critical criteria for the identification of keystone species in the overall co-occurrence network (16).

Except for each node, the topological properties were also calculated in the whole co-occurrence network to further understand the complex web of interconnected bacteria (15). These topological indexes contained the number of nodes, the number of edges, the average degree, the average shortest path length, the average connectivity, and the average clustering coefficient (Table S3).

The "Smallworldness" Index, another important index that represents the correlation compactness of individuals in a network, was computed based on the transitivity (any pairs of nodes with direct or indirect connections could be transitive) and the average shortest path length (the average number of steps along the shortest paths for all possible pairs of network nodes) in the "qgraph" R package. A network can be recognized as "small-world" if its "smallworldness" is higher than one (a stricter rule is "smallworldness" ≥ 3) (17). The judgment criteria of "small-world" network is to determine if it has a transitivity that is substantially higher than that of comparable random networks and an average shortest path length that is similar to or higher (but not many times higher) than that computed on random networks (18). Edge weights, signs, and directions are ignored in the computation of the indices.

S1.5.4 Estimation of the Richness of Global Microbiomes. Scaling laws describe the functional relationship between two physical quantities, i.e., the total abundance (N_T) and the abundance of the most abundant species (N_{max}), that scale with each other over a significant interval, underpin unifying theories of biodiversity, and are among the most predictively powerful relationships in biology. We used scaling laws to predict global airborne bacterial richness (S) based on the lognormal species abundance model (19). S could be predicted in terms of N_T, N_{max}, and the assumption that the rarest species is a singleton ($N_{min} = 1$), and by using the following equation:

$$S = \frac{\sqrt{\pi}}{a} exp\left\{\left[alog_2\left(\sqrt{\frac{N_{max}}{N_{min}}}\right)\right]^2\right\}$$

where a could be numerically solved by the following:

$$N_T = \frac{\sqrt{\pi N_{max} N_{min}}}{2a} exp\left\{\left[alog_2\left(\sqrt{\frac{N_{max}}{N_{min}}}\right)\right]^2\right\} exp\left\{\left[\frac{\ln(2)}{2a}\right]^2\right\}$$

$$\left\{erf\left[alog_2\left(\sqrt{\frac{N_{max}}{N_{min}}} - \frac{\ln(2)}{2a}\right)\right] + erf\left[alog_2\left(\sqrt{\frac{N_{max}}{N_{min}}} + \frac{\ln(2)}{2a}\right)\right]\right\}$$

The total airborne bacterial abundance was estimated using the qPCR quantification results of 16S rRNA gene copies from this study and published data. Thus, the N_T (global airborne bacterial abundance in the troposphere) is about 2.69 × 10^{25}. Details of the calculation process are given below. In addition, N_{max} was inferred based on the proportion of the typically most abundant genus or using the dominance-abundance scaling law:

$$N_{max} = 0.4 \times {N_T}^{0.93}$$

The most dominant taxonomic unit (based on a 97% 16S rRNA sequence similarity) in the troposphere was typically predicted to be a member of the *Bacillus* genus and accounted for around 1.2% of 16S rRNA gene reads in the whole dataset. N_{max} would then be approximately 3.2 × 10^{23}, which was close to the prediction by the scaling law, N_{max} = 1.78 × 10^{23}. The same method was also applied to microbial communities in global soil, global freshwater, and global leaf surfaces.

The approximate values of N_T and N_{max} for global microbiomes were calculated as follows:

(1) Troposphere. The exact upper boundary of the atmosphere (biosphere) was a Gordian knot for estimating the total number of airborne bacteria in the whole biosphere. The traditionally cited highest altitude for aerobiology is 77 km, due to the detection of microbes on the surface of one rocket; however, many researchers doubted that the microbes came from the rocket itself (in that study there was no detailed description of sterilized operations or of the steps taken to prevent contamination) or from the flying soil caused by the rocket making landfall (20). Nevertheless, it is certain that the troposphere contains approximately 80% of the total mass of the atmosphere; also, the temperature was -56°C and the humidity was nearly zero at the frontier between troposphere and stratosphere, where microbiomes can hardly survive (21). In addition, human activities mainly proceeded in the troposphere. As a result, our study focused on airborne bacterial communities in the troposphere, beginning at the land surface and extending to between 17 km at the equator and 7 km at the poles, with a mean altitude of 12 km (21). First, we assumed that the earth was a sphere with a radius of 6,371 km, and overlooked surface effects such as mountains and valleys. Second, we divided the troposphere into three circles based on the elevation: 0-1 km, 1-8 km, and 8-12 km According to the qPCR quantification results of the 16S rRNA gene copy from this study and published data, we predicted the mean bacterial density in the three circles: 4.8×10^5 cells/m^3 (0-1 km); 6.8×10^5 cells/m^3 (1-8 km); and 5.1×10^3 cells/m^3 (8-12 km) based on the measurements in this study and published studies (locations of these air samples can be found in Fig. S11). We assumed that the air was kept in the same conditions within cycles, ignoring the intra-circle variations in temperature and air pressure. The total bacterial loading in each circle was calculated by multiplying the total air volume and the mean bacterial density in the corresponding circle. Finally, we added them up to denote the total abundance of airborne bacteria in the troposphere. As a result, the N_T (global airborne bacterial abundance in the troposphere) is about 2.69×10^{25}, and N_{max} would be inferred to be 3.2×10^{23} or 1.78×10^{23} respectively according to the proportion of the most dominant genus and the scaling law (19).

(2) Global soil. The most dominant genus-level candidate (based on a 97% 16S rRNA sequence similarity) in global soil is the *Mycobacterium*, with an estimated proportion of 0.61% in each sample on average (22). As for the total number of bacteria cells in the global soil, we used data from the literature indicating that there are about 9.4×10^{28} microbial cells in global soil ecosystems (23). The detailed steps in determining the total number of bacterial cells in the global soil were as follows: First, the global soil was categorized into 12 classes according to types of ecosystems, and the total area on the Earth's surface was calculated. Then, mean bacterial densities with different depths (0-1 m and 1-8 m) were calculated based on as many measurements made around the world as possible. Next, boreal forest and tundra and alpine soils were assumed to be 1 m deep, but other classes of soil were 8 m deep. The soil volume could be calculated by multiplying surface areas and depths. The total number of bacterial cells in the global soil was estimated by the sum of the results from 12 classes of soil. The N_{max} of the global soil would be approximately 5.7×10^{26}, which is close to the estimated value of N_{max} using the dominance scaling law (19), 3.5×10^{26}.

(3) Global freshwater. We used the value 4.7×10^{25} as the number of microbial cells in global freshwater, including both rivers and lakes (23, 24). In addition, the most abundant taxonomic unit (based on a 97% sequence similarity in 16S rRNA reads) in global freshwater is typically a member of the *Pseudomonas* genus, and accounted for around 1.57% of the 16S rRNA gene reads in a sample based on the EMP database (25); the N_{max} of the global freshwater would then be 7.4×10^{23} or be estimated by the scaling law (19) as 3.0×10^{23}.

S1.5.5 Interactions of the Bacterial Community Composition of Air with Other Bacterial Habitats. The bacterial abundance table used in this study, containing 5,166 global samples from multiple habitats, was obtained from the EMP database (25). We also followed the unified standard workflow employed by the EMP to analyze our airborne bacterial sequence data based on a closed reference against Greengenes 13.8 in Qiime2 (26). The processed airborne bacterial OTU table was merged with the EMP OTU table, including samples collected from soil, rhizosphere, freshwater, ocean, air, human, and animal-associated habitats. The non-metric multidimensional scaling (NMDS) analysis was first performed based on the Bray–Curtis dissimilarity matrix for a comparison of microbial community compositions across habitats. In addition, the derived OTU table was used as the input file to estimate the proportion of each airborne bacterial sample attributable to various habitats on the genus level by using "SourceTracker" (27). To explore the patterns of interconnection and the coexistence of bacterial communities across various habitats at the global scale, the Earth's metacommunity co-occurrence network consisting of a communal catalog was also constructed with robust correlations ($\rho > 0.7$, p-*value* < 0.01).

S1.5.6 Quantifying the Relative Importance of the Microbial Community Assembly Process. A phylogenetic-bin-based null model analysis (iCAMP) was used to quantify the microbial community assembly process (28), which included three major steps: phylogenetic binning, conducting a null model analysis within each bin, and integrating the results of different bins to assess the relative importance of each process. Based on iCAMP, we were able to obtain quantitative results on community assembly processes containing heterogeneous selection (HeS), homogeneous selection (HoS), dispersal limitation (DL), homogenizing dispersal (HD), and drift (DR) from the statistical perspective.

S1.5.7 Multivariate Analysis. PCoA was used to visualize spatial and temporal differences in the keystone and core bacterial communities between the samples, based on the "Euclidean" index, and to evaluate the impacts of some environmental variables on these communities. We also used redundancy analysis (RDA) to identify the relationships between keystone or core bacterial genera and soluble ions or heavy metals in $PM_{2.5}$. This was performed by the "vegan" R package.

To quantify the relative contributions of the environment effect (separately or jointly) versus the distance effect on β-diversity, a variance partitioning analysis (VPA) was performed based on the RDA algorithm. We first selected a subset of explanatory variables from the set of all variables for constrained ordination, the goal of which was to reduce the number of explanatory variables entering the analysis while keeping the variance explained by them to the maximum. The standard method is stepwise selection, the combination of two approaches: forward selection (adding explanatory variables one by one) and backward selection (starting from the full model and deleting variables of which the least decreases the total explained variance). In addition, the initial set in our study included three explanatory groups: air quality (*e.g.,* AQI, PM_{10}, $PM_{2.5}$, NO_2, SO_2, and CO), meteorological conditions (*e.g.,* air temperature, air pressure, wind speed, wind direction, and relative humidity), and land cover type (*e.g.,* water/sea, forest, shrubs, grassland, cropland, and built-up areas). After a forward selection of environmental factors, the variables that remained for a VPA analysis were: NO_2, CO, PM_{10}, $PM_{2.5}$, wind speed, air temperature, air temperature, water/sea, cropland, and grassland (core bacteria); SO_2, NO_2, CO, O_3, PM_{10}, $PM_{2.5}$, air pressure, air temperature, relative humidity, wind speed, wind direction, water/sea, forest, shrubs, cropland, grassland, and built-up areas (keystone bacteria).

Moreover, to further explore the direct and indirect relationships among geographic locations, environmental variables, and bacterial communities, the structural equation model (SEM) was built

with the “lavaan” package (29). The prior model was set using all hypothesized reasonable indirect and direct links among the variables in SEM based on their pairwise correlations. We subsequently removed non-significant relationships and variables or created new links between other terms, *i.e.,* the post hoc model modification, until all quantitative indices met the overall goodness of fit. The composition of airborne keystone bacterial and core bacterial communities was indicated by their first principal coordinates (PCoA1). The SEM evaluation was based on the fit indices for the test of a non-significant chi-square test ($p > 0.05$), the root means square error of approximation (RMSEA) < 0.08, the standardized root means square residual (SRMR) < 0.05, the Tucker-Lewis index (TLI) > 0.90, and the comparative fit index (CIF) > 0.95.

S2. SUPPLEMENTARY RESULTS

S2.1 Small-world Characteristics in the Global Air, Soil, and Marine Bacterial Co-occurrence Network

In general ecology, real networks, including biological networks, have been proven to have the "small-world" property (30), which means that individuals are more connected to each other than in a random network. In this first attempt to construct an airborne bacterial network, we also computed the "smallworldness" index (17) by relying on the global transitivity of the network (31) and its average shortest path length. This indicated that the global airborne bacterial community network was not a "small-world" network ("smallworldness" index = 0.51 < 1) (Fig. 1*E*). In other words, the common "small-world" network in real ecosystems was not observed in our dataset. Conversely, the two other global bacterial datasets on topsoil and marine ecosystems both met the properties of a "small-world" network (soil "smallworldness" index = 5.82 > 3 for a stricter rule; marine "smallworldness" index = 1.21 > 1 for the general rule). The "smallworldness" index of the bacterial community network showed a decreased gradient from soil, marine, to air habitats, which was consistent with the variations in other topological properties (Table S3), such as the average shortest path length (3.03 < 3.97 < 5.24), diameter (9 < 10 <15), and clustering coefficient (0.48 < 0.58 < 0.67). Furthermore, the higher "smallworldness" index and shorter average shortest path length indicated a closer relationship between OTUs. This could be interpreted as increasing the speed of the response of the network to perturbations, finally leading to a more stable community network structure. All of the above results indicated that the airborne bacterial community network was not as stable as the soil and marine ecosystems and could be more easily affected by external factors.

S2.2 Composition and Structure of Airborne Bacterial Communities

S2.2.1 Differentia of Taxonomic Composition in Whole, Core, and Keystone Bacterial Communities on a Global Scale. The structure of the airborne bacterial community exhibited great dissimilarity from those of other ecosystems (Fig. 3*B*); in essence, a core set of 24 bacteria and 19 keystone species was exclusively determined to exist in the unique and huge airborne bacterial communities (Tables S1 and S4). In addition, OTU22, OTU94, and OTU159 overlapped both core bacteria and keystone species (Fig. S4), showing the great importance of whole communities due to hyper dominance and tight connections with other members. These could be recognized as the top three key species. What they have in common is that they are all gram-positive bacteria and belong to the same phylum, *Actinobacteria*.

In addition, the differences in the community composition structures of keystone, core, and all OTUs were further compared. As one of the most widespread phyla in airborne bacterial communities (24.8% in whole communities), *Firmicutes* has been well documented as producing endospores that are resistant to extreme desiccation for surviving in extreme conditions. However, no members of *Firmicutes* were identified as keystone species because there were few interactions with communities. By contrast, *Actinobacteria* did not exhibit overwhelming superiority with regard to abundance (18.1% in whole communities and 19.6% in the core set); yet they were closely related to the whole airborne bacterial communities and even exceeded *Proteobacteria*, as embodied in the finding that *Actinobacteria* made up a great share (72.2%) of keystone communities (Fig. S4 and Table S4). In summary, the composition of the whole global airborne bacterial community was similar to that of the core set, while both displayed wide variations from keystone communities (Fig. S4).

S2.2.2 Keystone Taxa Associated with Evolutional and Ecological Functions. According to the rich-gets-richer preferential attachment process of growth in a scale-free network (32), the highly connected nodes, namely, the keystone species, could acquire more links, contributing to the establishment of the whole microbial network, which means that keystone nodes are recognized as initial components in networks. Thus, in evolutionary terms, this suggests that keystone species emerged earlier than other species, and that their lineages might have a longer evolutionary history

in microbial co-occurrence networks (15). This has important implications for exploring the origins of microbes in the atmosphere and even in other ecosystems. For example, an important keystone taxon, *Frankiales* (OTU19 & OTU22 in Table S4), was assumed to have an adaptable ancestral bacterium that evolved to occupy many different ecological niches, including the root nodules of woody dicots, hot springs, rocky surfaces, gamma-irradiated substrates, activated sludge, compost, and soils (33), which could be assumed to be the crucial ancestor of many airborne bacteria. In addition, the close relationship of *Frankiales* with soluble ions in RDA also revealed its wide adaptation (Fig. S12).

The keystone concept was derived from food-web ecology (34), while the interspecies relationships, including mutualism, commensalism, parasitism, competition, and others, were mainly manifested as trophic relationships. Thus, the functions of most keystone species were related to nutrition and metabolism, which was also reflected in our study. For instance, *Rhizobiales* (OTU63 & OTU55 in Table S4) occupies a unique physiological function (biological nitrogen-fixation) and has been recognized as key to the global cycling of nitrogen (35). Similarly, *Burkholderiaceae* (OTU250 in Table S4) can produce secondary metabolites and significantly affect microbial interactions in the network (36), and *Gaiellales* (OTU372 & OTU721 in Table S4) can utilize several organic compounds (37) and plays an important role in the whole process of the cycling of nutrition in microbial communities. The integrity of the overall community and its unaltered persistence through time, namely its stability, have been authenticated to be profoundly affected by the activities and abundance of these individual populations, which make up the keystone set (38).

Moreover, for insight into the functions of keystone species, the microbial keystone taxa in various ecosystems were also summarized from other studies (Table S5). The point worth emphasizing here is that diverse members of the *Rhizobiales* and *Burkholderiales* orders have been consistently recognized as keystone taxa in various ecosystems and habitats, from soil to aquatic systems and from equatorial to polar habitats (Table S5). *Rhizobiales* contains not only nitrogen-fixing bacteria (*Rhizobium* spp. and *Bradyrhizobium* spp.), but also methanotrophs (*Methylobacterium*) with high abundance in the phyllosphere (35). In addition, *Burkholderiales* includes several well-known pathogenic bacteria, such as *Bordetella*, *Ralstonia, Oxalobacter,* as well as *Burkholderia*, one of the most versatile and virtually ubiquitous terrestrial microbial groups (36). Although *Rhizobiales* and *Burkholderiales* have been well documented as playing an important role in keystone bacterial communities in the natural world, the computational identification results have shown that not all of their members could be considered keystone taxa. Plenty of its subordinate taxa in the two orders had no significant impacts on community composition or function. Their common keystone roles, which occur in diverse habitats, might also be caused by their large abundance and wide occupancy in natural environments. Despite that, the possibility is still high that members of *Rhizobiales* and *Burkholderiales* could become keystone taxa, and future studies should further evaluate their roles as potential keystone taxa in microbial functions and interactions.

S2.2.3 Airborne Bacterial Diversity Pattern in Mid-latitudinal Regions. The global latitudinal diversity pattern in whole airborne bacterial communities showed a clear trend of richness peaking at intermediate latitudes and declining towards the equator and the two poles (Fig. 2*A*). However, there were still some dots that deviated from the fitting line in the above latitudinal pattern, especially in the mid-latitudinal regions (35° - 45°). In order to explain the unknown large deviation, mid-latitudinal samples ($n = 64$) were selected for further study. We discovered that the bacterial richness strongly correlated with both PM_{10} concentration ($R^2 = 0.549$, $p < 1 \times 10^{-10}$) and $PM_{2.5}$ concentration ($R^2 = 0.517$, $p < 1 \times 10^{-7}$) (Fig. S13). The richness was higher in moderately polluted air, but lower with good air quality and heavy pollution, which might be attributed to the fact that pollutants could also be considered a source of nutrients for the microbiome. In addition, populations are distributed unevenly across the globe, a situation that is much more obvious in mid-latitudinal regions. In the more developed cities and economic circles, populations are much more dense, while they are sparsely populated in deserts, polar regions, and high mountains. For instance, Hong Kong is densely populated, with 6,544 people per square kilometer. Its population density is far above that of Mt. Ailao, which is at a similar latitude. However, there was no significant

difference in richness between the two sites. In conclusion, from a global perspective, airborne bacterial diversity follows a downward opening parabola-shaped latitudinal pattern and is hardly influenced by human distribution and the intensity of human activities.

S2.3 Impacts of Urbanization on Airborne Bacterial Genotypes

The structure (*i.e.,* cell shape and cell management) of airborne bacteria changes a great deal due to urbanization, as can be seen from the finding that the percentages of *Bacilli* and of bacteria existing in clusters in urban areas were higher than those in terrestrial background and offshore areas (Fig. S14). Although air temperature is mainly driven by latitude, the relatively higher air temperature in urban areas than in terrestrial background and offshore areas in general caused there to be a correspondingly higher optimal temperature range for airborne bacteria in urban areas (Fig. S15*a*). For instance, the relative abundance of thermophilic bacteria was higher in the urban areas of Guangzhou (5.25%) than in a background area with a similar latitude, Mt. Ailao (2.39%). Nevertheless, tolerance of airborne bacteria to the environment did not change with urbanization; concretely, no difference was observed in the environmental resistance caused by sporulation and the surviving modes (symbiotic or free-living) of bacteria between urban and background air (Figs. S15 *b* and *c*). Similarly, no great difference was found in the ratio of gram-positive to gram-negative airborne bacteria (and pathogens) in urban, terrestrial background, and offshore areas, further illustrating that urbanization did not change the likelihood of human illness related to Gram- and/or Gram+ pathogens (Fig. S16). The motility of airborne bacteria in urban areas was higher than in natural areas (terrestrial background and offshore areas), aligning with the ratio of bacteria carrying flagella. This might enlarge the spread of airborne pathogens and further increase the likelihood of pathogenic infections in cities (Fig. S17).

S2.4 Environmental Impacts on Global Airborne Bacterial Communities

S2.4.1 Relationships between Environmental Factors and Airborne Bacterial Diversity and Biomass. The variations in the diversity of global airborne bacteria with latitude were similar to that in the AQI index scores and concentrations of PM_{10}, $PM_{2.5}$, SO_2, and CO, and were also related to other meteorological parameters such as air temperature, air pressure, wind direction, wind speed, and relative humidity (Fig. S5). The pairwise correlation analysis suggested that the relationships of richness with other geographic locations, namely altitude ($R^2 = 0.019, p = 0.209$) and distance to coast ($R^2 = 0.019, p = 0.209$), were either weak or uncorrelated. Besides, different pollutants showed different relationships with bacterial diversity, mainly classified as parabolic fitting curve associations (AQI, $PM_{2.5}$, and PM_{10}), positive correlations (SO_2, and CO), and independent relationships (NO_2, and O_3). Among the above meteorological conditions, the best-predicted factor of bacterial richness was relative humidity ($R^2 = 0.190, p < 10^{-7}$).

The correlations between the total airborne bacteria biomass (the number of copies of 16S rRNA genes) and environmental factors were also analyzed (Fig. S7); however, three factors were significantly related to bacterial biomass: concentration of NO_2 ($R^2 = 0.109, p < 10^{-5}$), wind speed ($R^2 = 0.173, p < 10^{-8}$), and wind direction ($R^2 = 0.189, p < 10^{-6}$). According to the parabolic fitting curve relationship, the biomass was much higher with a southerly wind blowing than with winds blowing from other directions. At this point, we originally hypothesized that warm temperature and low latitude were hospitable to bacterial survival and diversity, because most samples were collected in the northern hemisphere, and southerly winds could increase the local air temperature to a certain extent. However, this hypothesis conflicted with another discovery that there were no relationships between biomass and either latitude or air temperature, so the hypothesis was soon disavowed. As a result, there must be some unknown mechanisms of wind direction driving airborne bacterial biomass, which might be related to atmospheric circulation or other geographic effects on microbial communities.

S2.4.2 Environmental Filtering Impacting Each OTU in Whole Global Airborne Bacterial Communities. To further understand the impacts of environmental factors on whole airborne bacterial communities, we examined one by one the correlations between 11 typical environmental

variables with a total of 10,897 OTUs and drew up a summary. More than half of the OTUs (57.7%) showed no connections with the environmental variables. Also, among the few existing relationships, the degree of correlation of the majority (97.4%) was not high (*absolute Spearman's* $\rho < 0.5$), indicating that the impacts of environmental filtering on the abundance of each specific OTU were weak (Fig. S18*a*). In addition, the total number of significant correlations of each OTU with air pollutants ($n = 6,440$) was much larger than with meteorological conditions ($n = 2,284$), particularly for CO, PM_{10}, and $PM_{2.5}$, which were respectively correlated with 14.4%, 12.9%, and 12.5% members of whole airborne bacterial communities (Fig. S18*b*). Moreover, most of the impacts of air pollutants on airborne bacteria (78.0% in keystone bacteria, 72.5% in core bacteria, and 69.0% in all OTUs) were positive (Fig. S18*c*), which indicated that it was easier for the structure of airborne bacterial communities to be positively influenced by moderate air pollution. Air pressure and relative humidity also contributed to shaping community structure and could directly affect 9.8% and 8.2% of bacteria, respectively. Other meteorological conditions, however, had almost no impacts on bacterial abundance.

Although environmental factors had less of an effect on the total biomass (Fig. S7), bacterial richness was associated with most air pollutants and climatic conditions (Fig. S5). This determined the patterns of diversity of airborne bacterial communities worldwide, although some of these environmental factors were inter-related (Fig. S6*a*). Abiotic factors, namely environmental variables, affected airborne bacterial richness in various regions but had less influence on the abundance of each specific OTU, with a low ratio (7.28%) of the number of existing significant relationships ($p < 0.05$) to the number of all possible connections between OTUs and environmental variables. Thus, there must be some unknown connections between the environmental variables and the overall community structure. Here, the significant effect frequencies of environmental factors on each core and keystone OTU were quantified by a multiple regression analysis, showing the ratios of 24.6% and 44.0%, respectively (Fig. S17*c*). This revealed that the strength of the impact of environmental filtering on these two crucial bacterial communities largely outweighed that of other normal OTUs. This suggested that keystone species and core bacteria were much more affected by the environmental filtering process and played a key role in shaping the composition of the whole community, which could be considered as one of the biotic factors shaping whole communities.

S2.4.3 Impacts of Environmental Variables on Keystone and Core Bacterial Communities. To investigate the temporal and spatial variability of the two crucial airborne bacterial communities (24 core OTUs and 19 keystone species), we analyzed their correlations with other potential environmental factors by multiple regressions. The results showed that all keystone species and most of the core bacteria were significantly ($p < 0.05$) correlated with at least one out of the 11 environmental factors (Figs. S19 *c* and *d*). VPA showed that air quality affected the keystone bacterial communities most and a subset of air pollutants including SO_2, NO_2, CO, O_3, PM_{10}, and $PM_{2.5}$ together explained 53.3% of the structural variations. This was substantially higher than the figures for meteorological condition (26.54%) and landscape coverage type (18.3%) (Fig. S19*f*). This result was consistent with the high frequency with which air pollutants were related to keystone bacterial communities (26.3%) and the comparatively low frequency with which they were related to meteorological conditions (17.7%) (Fig. S18). Regarding the core bacteria, the contribution of land coverage type (15.9%) was far lower than that of the other two groups (36.5% and 31.9%) (Fig. S19*e*). Notably, the three major groups had a significant impact on whole communities, explaining over 70% of the variation in both the core (70.6%) and keystone (80.1%) communities.

In addition, as well-known antimicrobial agents, heavy metals in $PM_{2.5}$ were also explored for their effects on bacterial communities. However, the heavy metals in the air showed little effect on temporal or regional variations in the structure of keystone and core bacterial communities, as reflected by the similar distributions of all samples (RDA, Fig. S12). As for species differentia, keystone taxa were all negatively affected by heavy metals, while core bacteria had various responses to heavy metals. For example, around half of core OTUs were positively correlated with heavy metals, probably because these abundant core bacteria were adaptable to atmospheric environments, while metal-induced toxicity was negligible. The remaining core bacteria showed

weak or negative correlations with heavy metals. The mechanisms of the metals affecting airborne bacteria were complex, and further studies involving discussions of specific conditions and toxicity experiments of cells are needed.

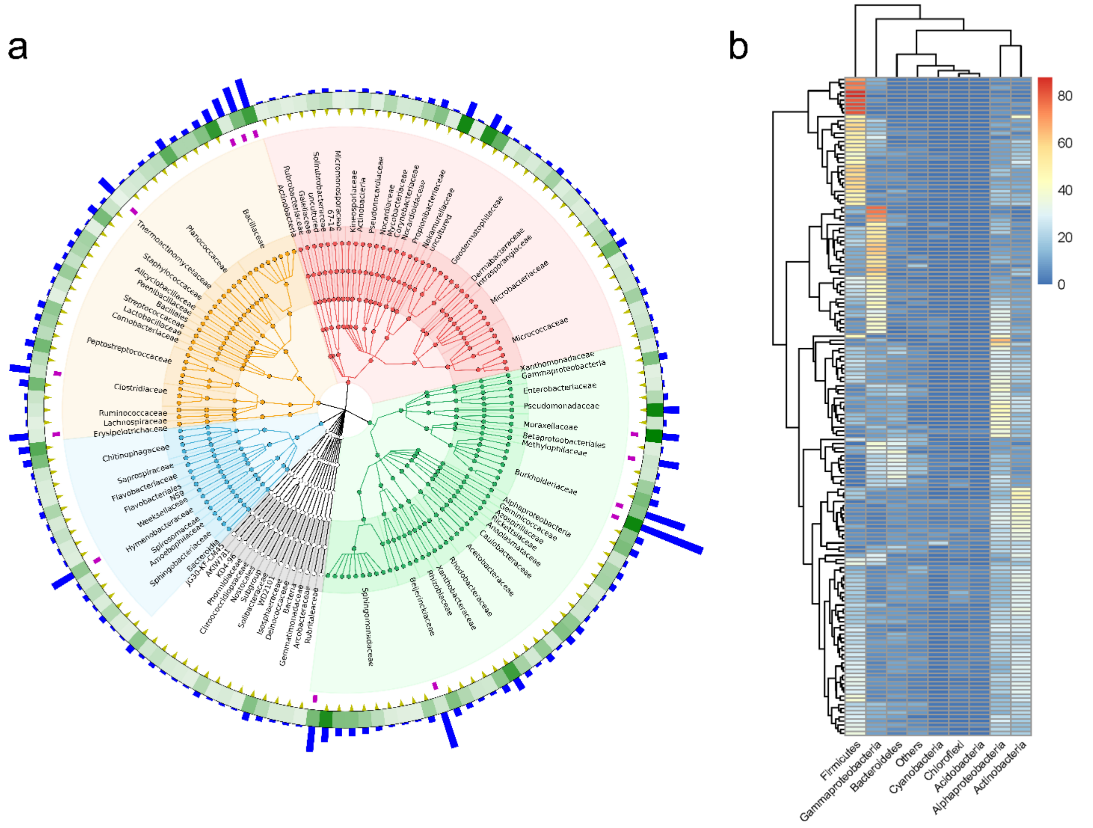

Fig. S1. Airborne bacterial abundance distributions across the globe (*n* = *370*). (*a*) Phylogenetic tree of dominant airborne OTUs. The center is a phylogenetic tree of bacteria abundant in the worldwide atmosphere. The middle ring corresponds to body sites at which the various taxa are abundant and color-coded at the phyla level. The airborne bacteria are dominated by four phyla: *Proteobacteria* (green), *Firmicutes* (yellow), *Actinobacteria* (red), and *Bacteroidetes* (blue). In the external middle ring, the most abundant bacteria (mean relative abundance > 1%) are indicated by purple rectangles, and the relatively inadequate bacteria (mean relative abundance < 1%) are indicated by yellow triangles. The heights of blue bars outside the circle correspond to the abundance of taxa at the body site of greatest prevalence. (*b*) Phyla-level (class-level for *Proteobacteria*) community compositions across the globe. Each column represents a phylum (sub-phylum). Each row represents a sample collected from various environments at different times, which are clustered based on the Bray-Curtis similarity of phylum-level compositions. The taxonomic annotation is labeled with text at the bottom. The relative abundance of each phylum in specific samples is depicted by the color of the box with reference to the legend (unit: %) at the upper right.

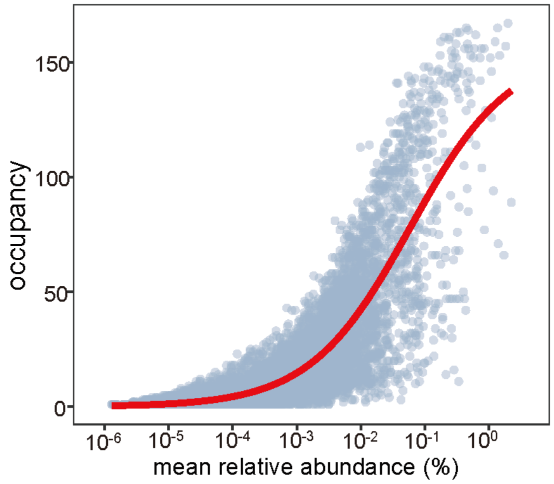

Fig. S2. Abundance-occupancy relationship (AOR): mean relative abundance (*x-axis*) and occupancy (*y-axis*) plot after combining the OTUs with the same annotation (*n = 10,897*). The mean relative abundances were estimated by averaging the relative abundances of each OTU in all samples; occupancy represents the number of samples in which the OTU was detected. The fitted model (sigmoid curve) was occupancy versus logarithm of abundance for each species, and the red solid line is the global fit to all species and samples.

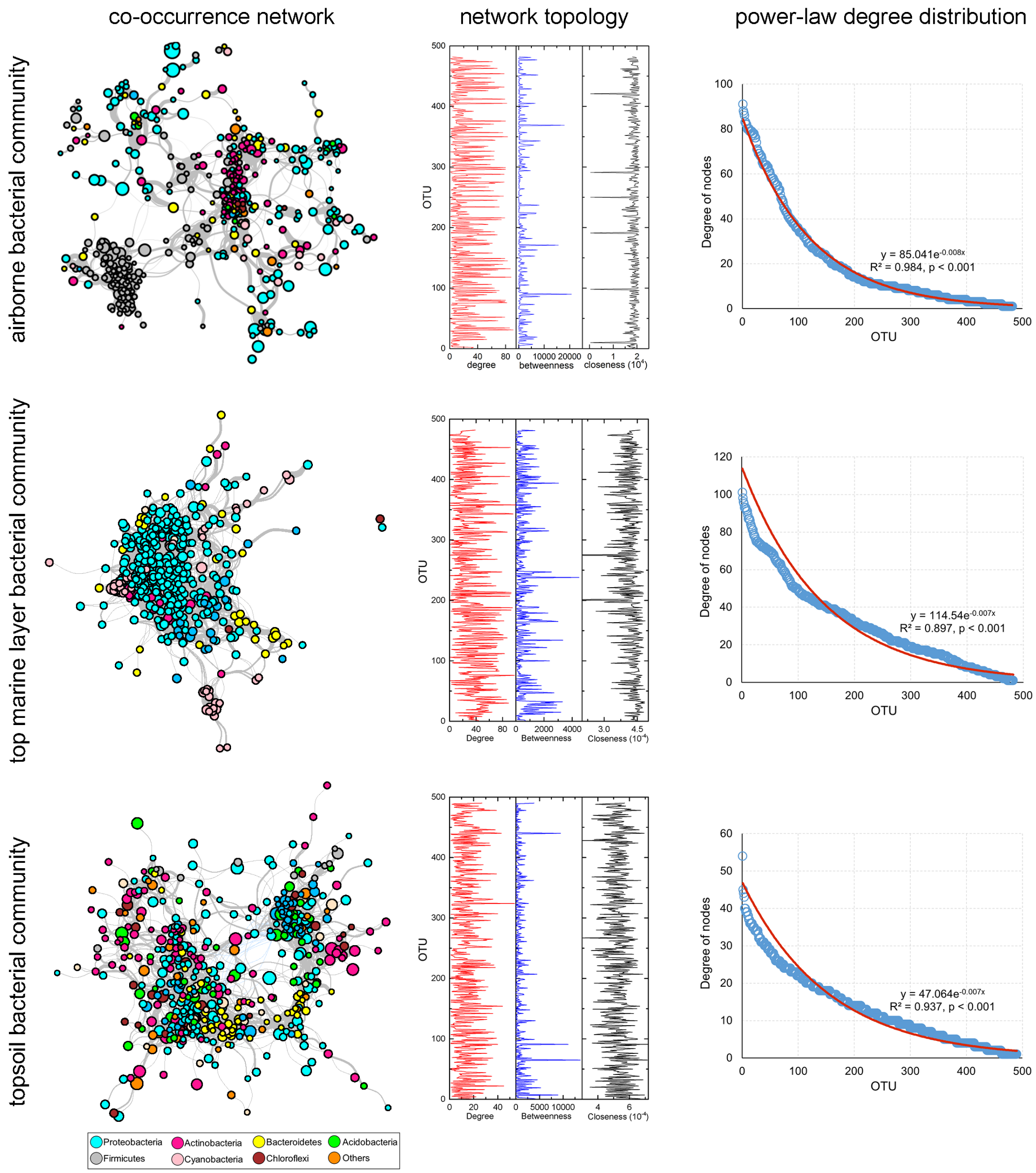

Fig. S3. Comparison of networked bacterial communities in the global atmosphere, topsoil, and top marine layer. Co-occurrence network: The connection (edge) stands for a strong (Spearman's ρ > 0.6) and significant (*P*-value < 0.01) correlation. The nodes represent the combined OTUs with a unique annotation for the genus level in the datasets. The size of each node is proportional to the mean relative abundance across all samples. Nodes are colored by the phyla of the bacteria. Network topology: The degree and centrality (betweenness and closeness) of each node from the networks were measured. Degree represents the number of direct connections of a node with other OTUs in the whole community. Betweenness centrality reveals the role of a node as a bridge between components of a network. Closeness centrality is a measure of the average shortest distance from each node to each other node. Power-law degree distribution: The node-degree distribution shows a power-law behavior for an airborne bacterial community co-occurrence network in airborne bacterial communities (R^2 = 0.984, p < 0.001), marine layer bacterial communities (R^2 = 0.897, p < 0.001), and topsoil bacterial communities (R^2 = 0.937, p < 0.001), respectively.

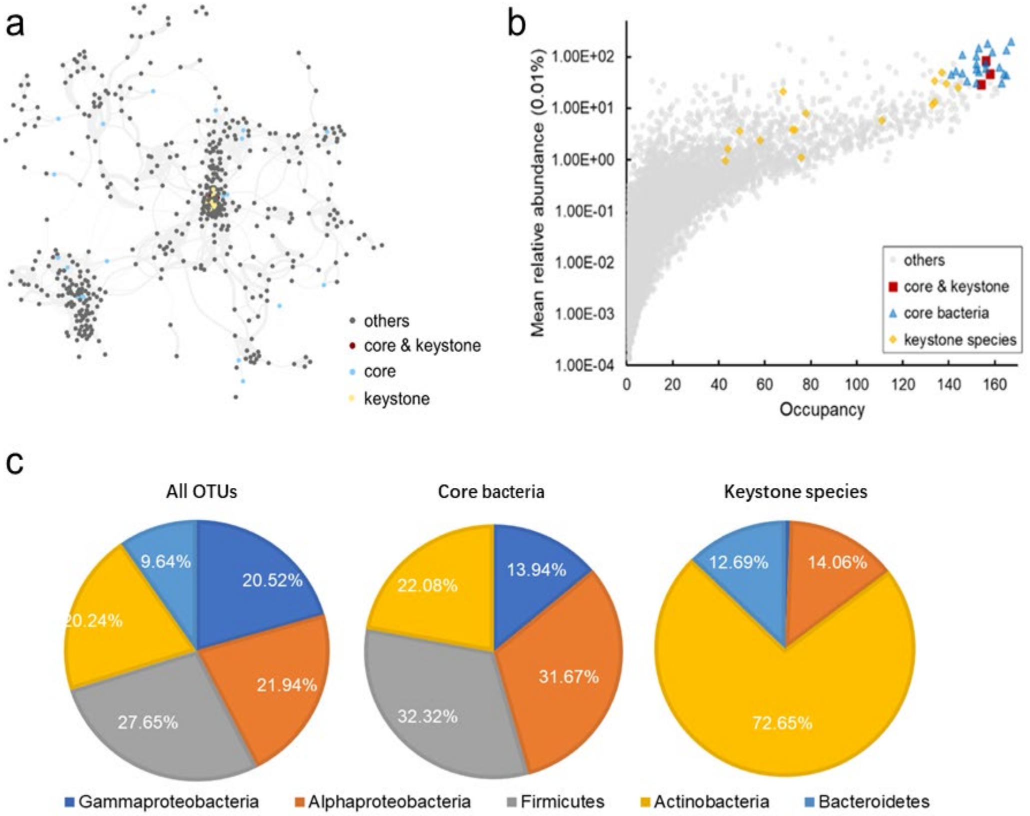

Fig. S4. Role and composition of the global-scale core and keystone bacterial communities in the atmosphere. (*a*) The position and role of core and keystone bacterial communities in a community network and (*b*) AOR pattern. (*c*) The taxonomic composition of bacterial communities at the phylum level (class level for *Proteobacteria*) for whole communities, core bacteria, and keystone species.

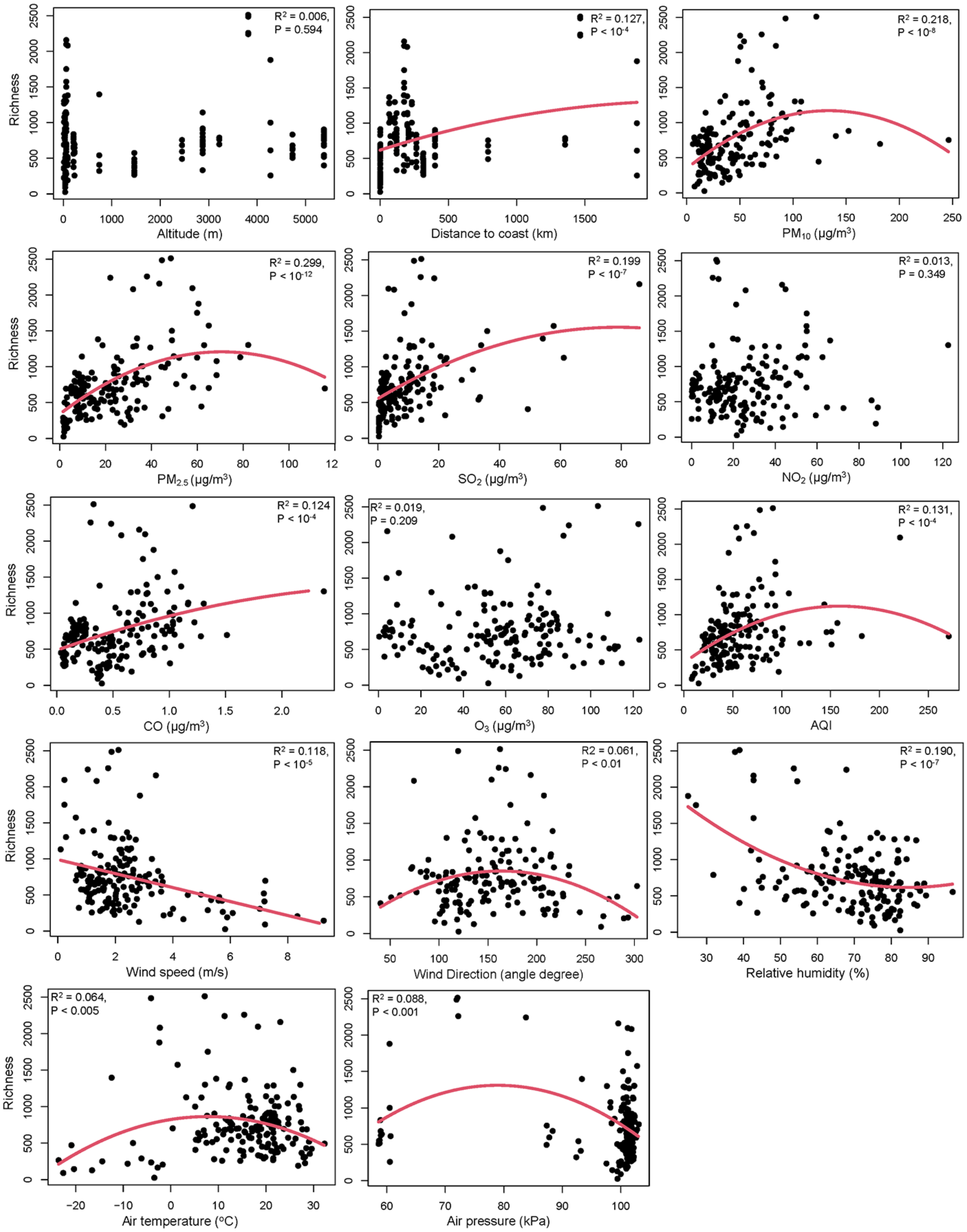

Fig. S5. Relationships of the global airborne bacterial diversity index (OTU richness) with air quality and meteorological conditions.

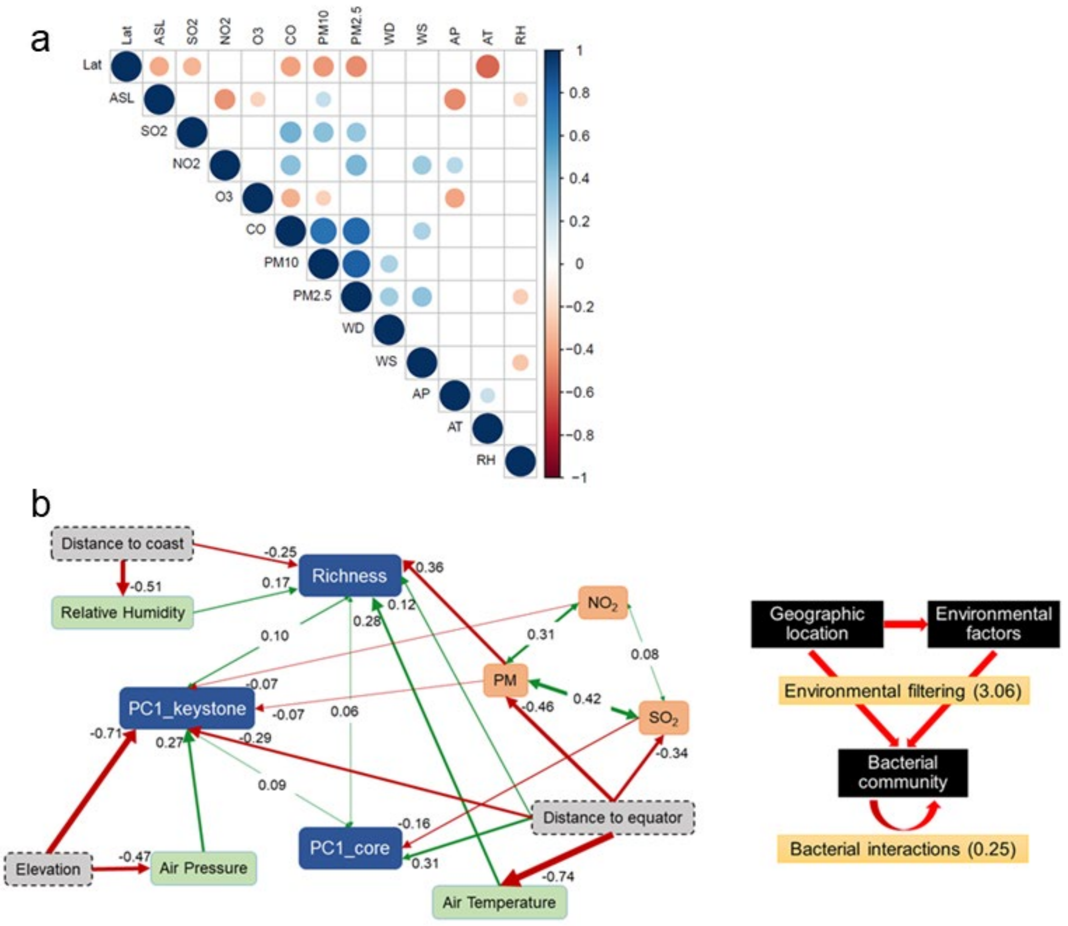

Fig. S6. Impacts of environmental filtering on airborne bacterial communities. (*a*) The correlation matrix among geographic locations, air quality, and meteorological conditions. The color gradient on the right indicates Spearman's rank correlation coefficients, with more positive values (dark blue) indicating stronger positive correlations and more negative values (dark red) indicating stronger negative correlations. The sizes of the colored circles represent correlation strengths. (*n = 370 biologically independent samples*). (*b*) SEM showing the direct and indirect relationships among geographic locations, environmental variables, bacterial interactions, and community composition. The diversity of global airborne bacteria can be explained by a combination of biotic and abiotic factors. The keystone and core bacterial communities are represented by the PC1 from the Euclidean similarity index principal coordinate analysis. "PM" represents the collected particle mass concentration of each sample. The one-way arrows represent causal relationships, while the two-way arrows represent mutual effects. The thickness of the lines shows the strength of the association (green – positive; red – negative). Standardized path coefficients (β) all appear near the corresponding pathways. The goodness of fit was acceptable: Model χ^2 = 23.008, d.f. = 16, P (chi-square test) = 0.114, root mean square error of estimation (RMSEA) = 0.049, Standardized Root Mean Square Residual (SRMR) = 0.028, comparative Fit Index (CFI) = 0.973, Tucker-Lewis Index (TLI) = 0.950, n = 370 biologically independent samples. The aligned right panel summarizes the total effects of environmental filtering and bacterial interactions on shaping communities.

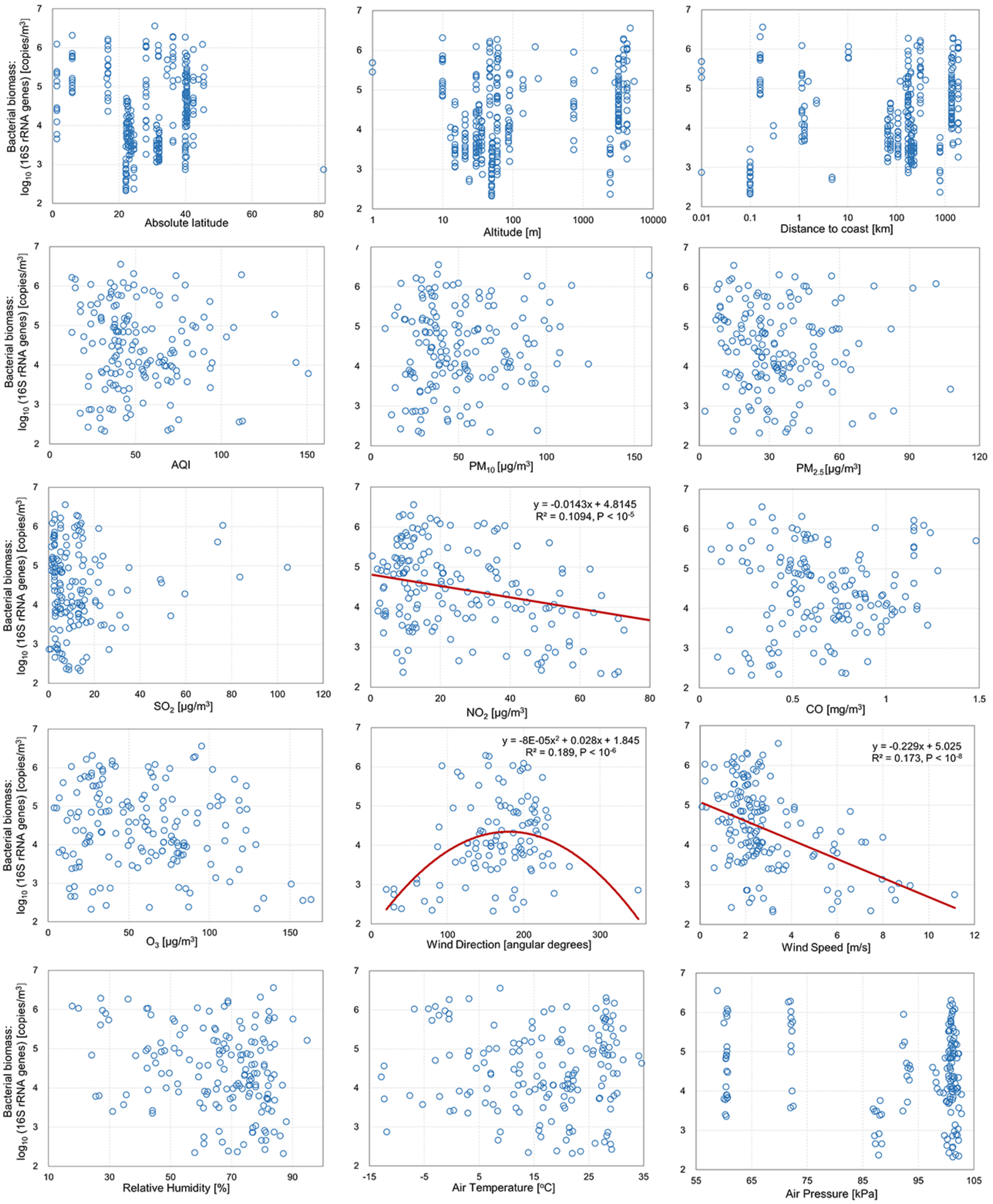

Fig. S7. Relationships of global airborne bacterial biomass (16S rRNA gene copy number) with geographic locations, air quality, and meteorological conditions.

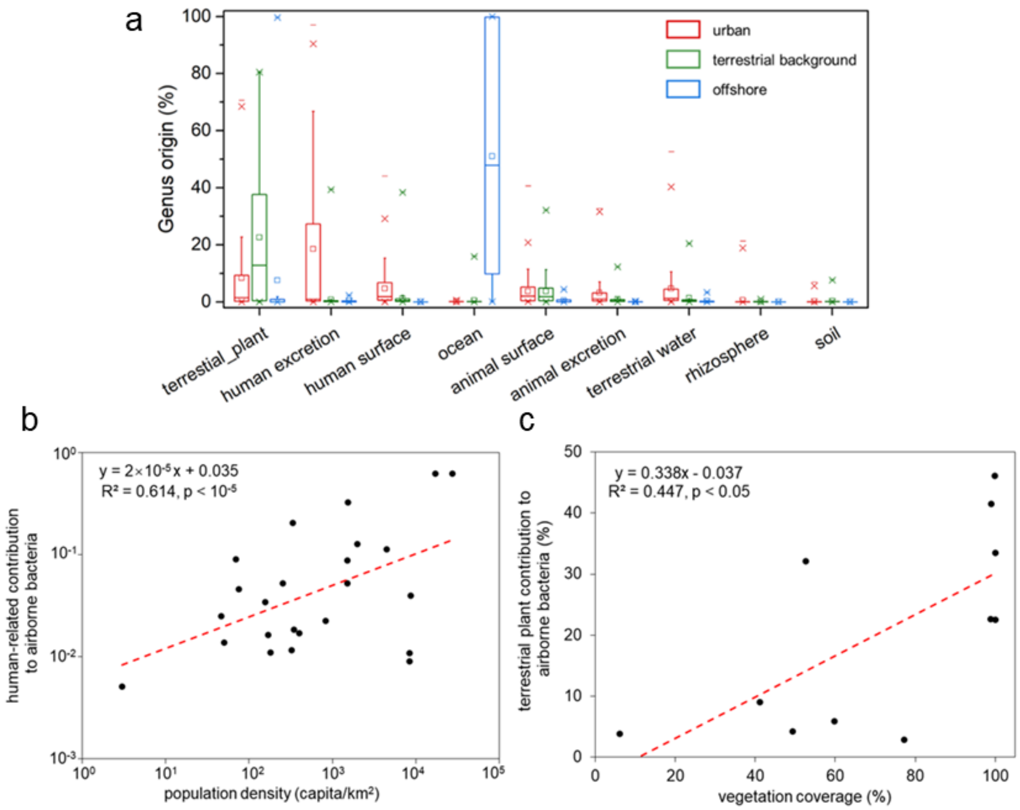

Fig. S8. Global airborne bacterial source contribution. (*a*) Potential contributions of various environments to airborne bacteria (at the genus level) in urban, terrestrial background, and offshore areas respectively at the global scale. (*b*) Relationship of the human-related contribution to airborne bacteria and population density in urban areas; relationship of the terrestrial plant contribution to airborne bacteria and vegetation coverage in background areas.

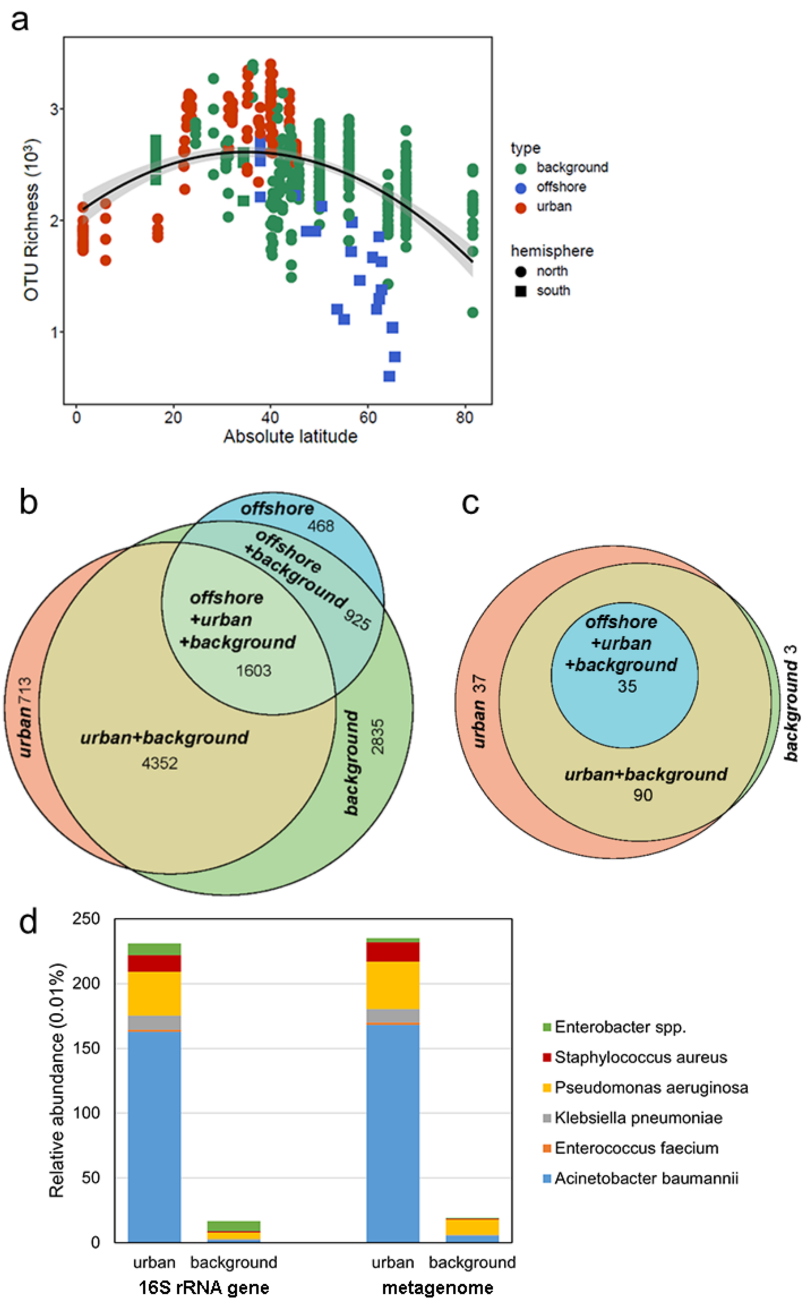

Fig. S9. Anthropogenic impacts on the composition of airborne bacterial and pathogenic communities. (*a*) Latitudinal distribution of airborne bacterial α-diversity (richness). Plotting OTU richness against the absolute latitude of sampling locations shows that there is no significant disparity in richness between urban and background areas. (*b*) Number of exclusive and shared airborne bacteria in urban, terrestrial background, and offshore areas. (*c*) Number of exclusive and shared airborne pathogens in urban, terrestrial background, and offshore areas. (*d*) Ratio of the abundance of airborne ESKAPE pathogens (*Enterococcus faecium, Staphylococcus aureus, Klebsiella pneumoniae, Acinetobacter baumannii, Pseudomonas aeruginosa, and Enterobacter* spp.) in urban areas to background areas based on two datasets, those of the 16S rRNA gene and metagenome, respectively.

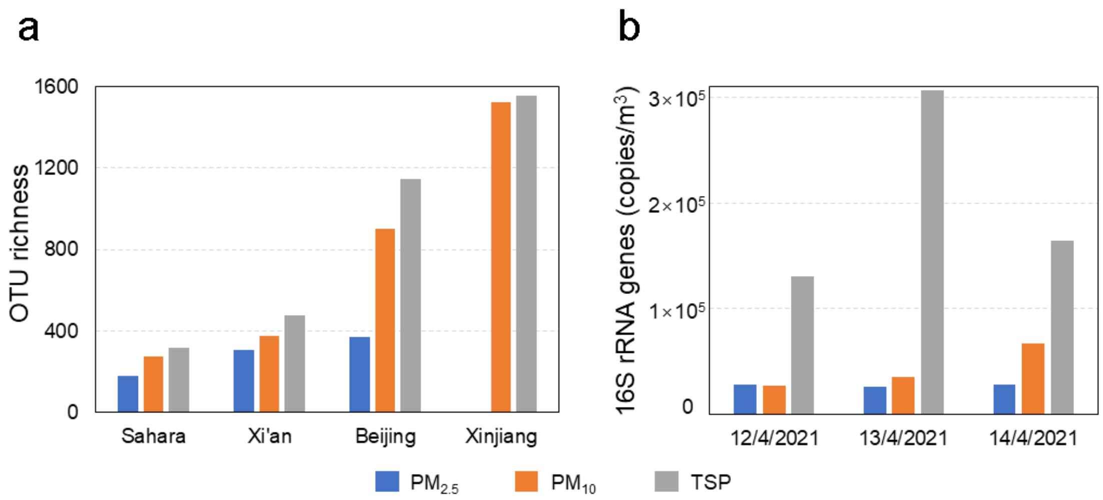

Fig. S10. Effect of particulate matter sizes on airborne bacterial richness and total biomass. (*a*) Comparisons of the airborne bacterial richness in $PM_{2.5}$, PM_{10}, and TSP collected in the same site during the same period. Data from the literature were drawn from References (39-42). (*b*) Comparison of the airborne bacterial biomass in $PM_{2.5}$, PM_{10}, and TSP collected in the hanging garden on the 11th floor of a twelve-story building at the Hong Kong Polytechnic University (22.31N, 114.18E) on three consecutive days.

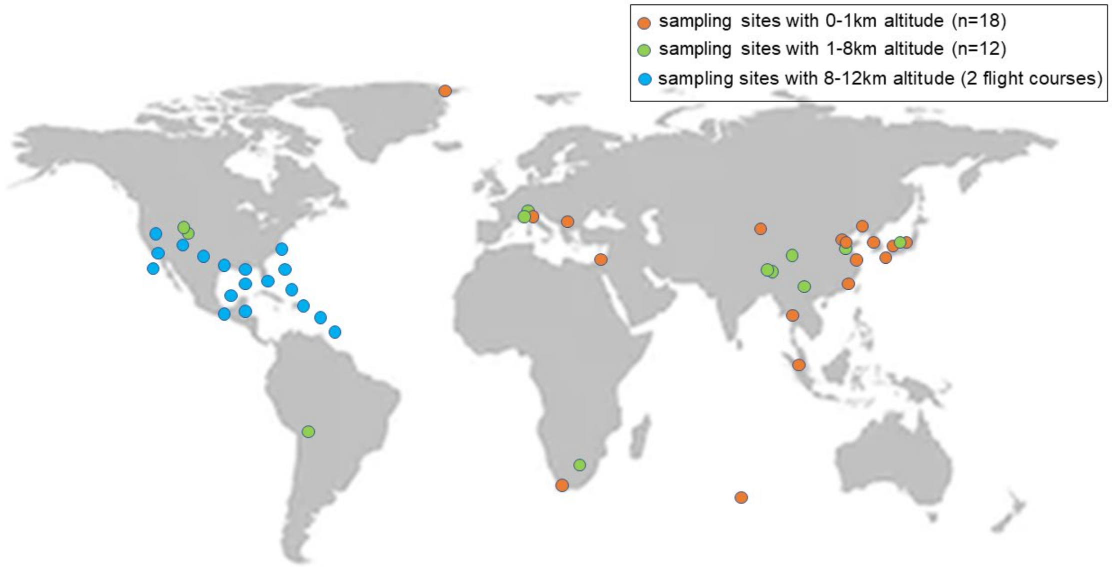

Fig. S11. Map showing the geographical locations of the sampling sites for estimating the mean airborne bacterial density from this study and other studies (42-64).

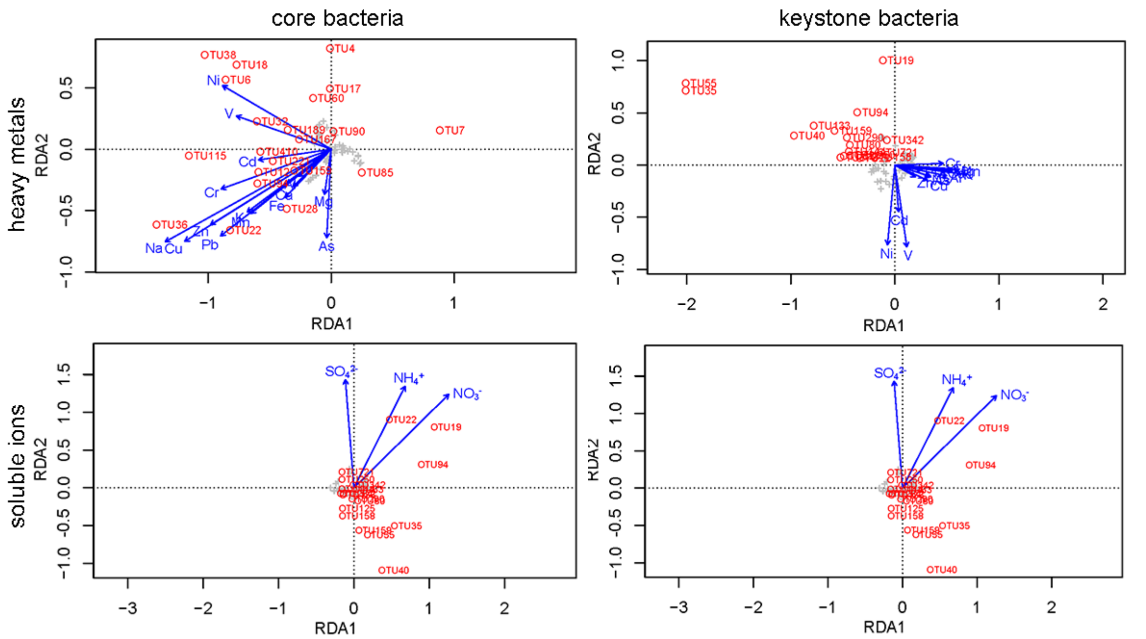

Fig. S12. RDA identifying the relationships between core & keystone bacteria and soluble ions & heavy metals in air samples. Blue arrows represent the environmental factors, with their length indicating the strength of the correlations between the environmental factors and the sample distributions. The longer the line is, the stronger the corresponding correlation will be. The included angle between the arrows denotes the correlation between environmental factors. An acute angle means a positive correlation, and an obtuse angle means a negative correlation. A smaller included angle corresponds to a stronger correlation. Red labels indicate the OTU number and gray markers indicate the distribution of the samples.

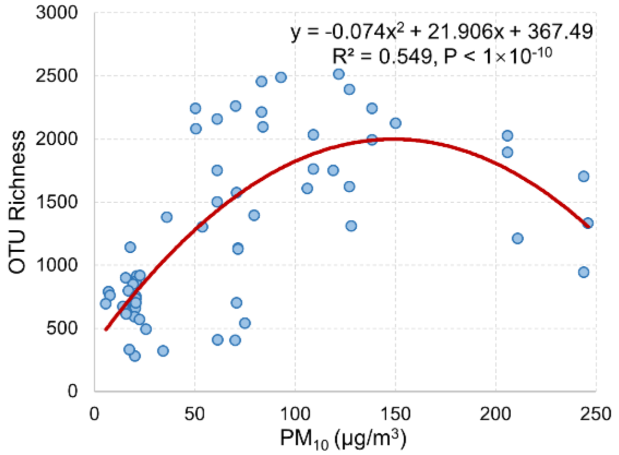

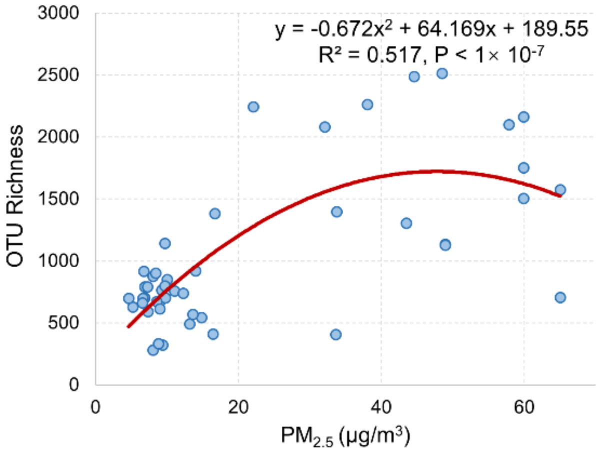

Fig. S13. Airborne bacterial richness (*y-axis*) and PM concentration (*x-axis*) plot of samples collected in the mid-latitudinal regions (35° - 45°). The richness was strongly correlated with PM_{10} concentration (*R^2=0.549, P < 1 × 10^{-10}*) and $PM_{2.5}$ concentration (*R^2=0.517, P < 1 × 10^{-7}*), explaining the large deviation from the fitting line of Fig. 2*A* in mid-latitudinal areas.

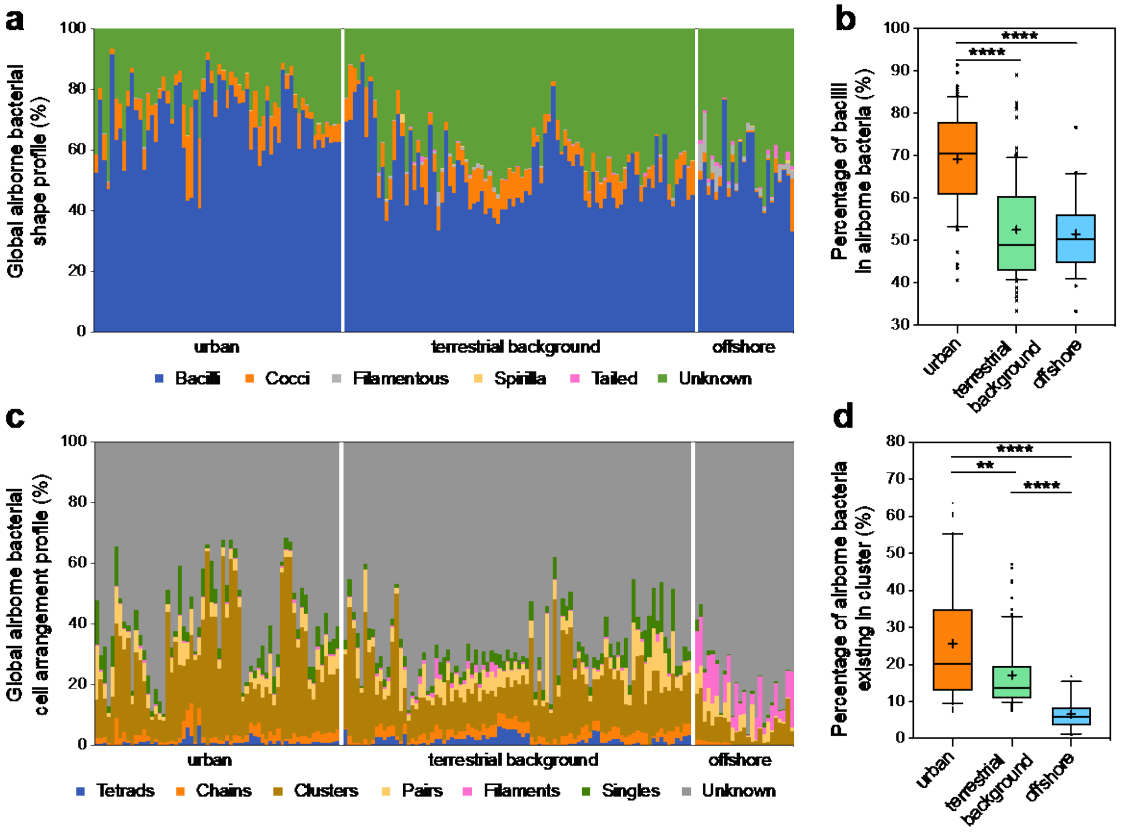

Fig. S14. Structure of global airborne bacterial cells in urban, terrestrial background, and offshore areas. (*a*) Global airborne bacterial shape profile. (*b*) Percentage of *Bacilli* in airborne bacteria. (*c*) Global airborne bacterial cell arrangement profile. (*d*) Percentage of airborne bacteria existing in clusters.

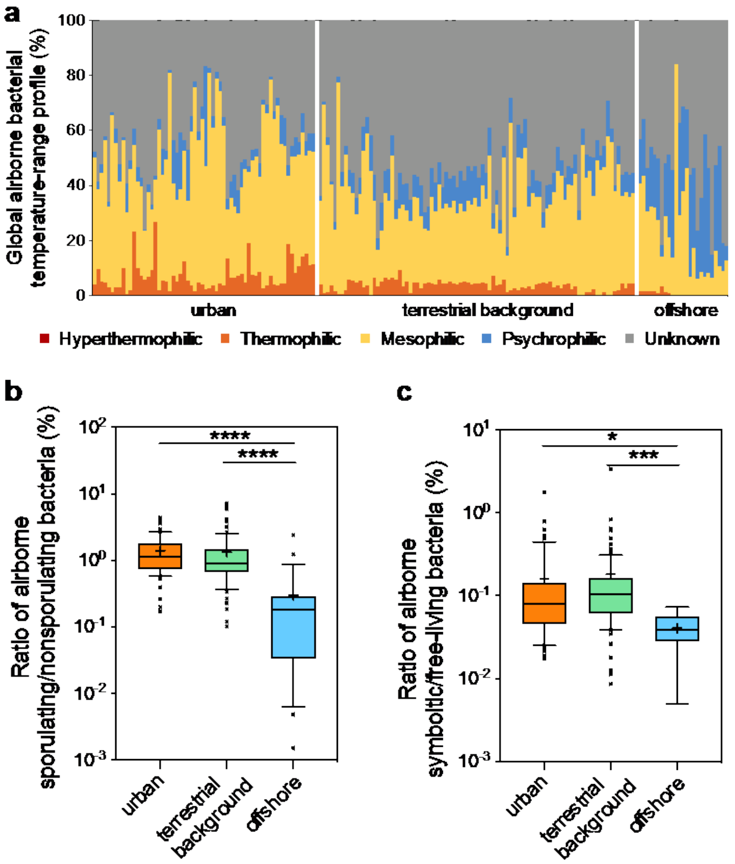

Fig. S15. Adaptation of global airborne bacteria to local environments in urban, terrestrial background, and offshore areas. (*a*) Optimal temperature range of airborne bacteria. (*b*) Sporulation of airborne bacteria. (*c*) Ratio of airborne symbiotic/free-living bacteria.

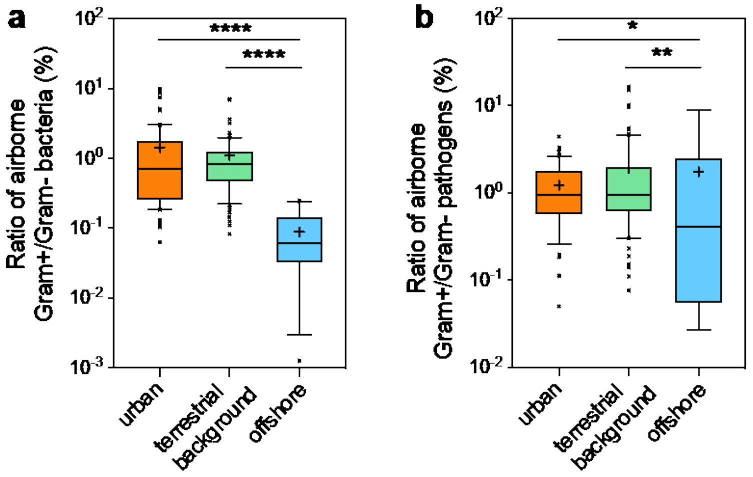

Fig. S16. Gram-positive (Gram+) and negative (Gram-) of global airborne bacteria in urban, terrestrial background, and offshore areas. (*a*) Ratio of airborne Gram+ to Gram- bacteria. (*b*) Ratio of airborne pathogenic Gram+ to Gram- bacteria.

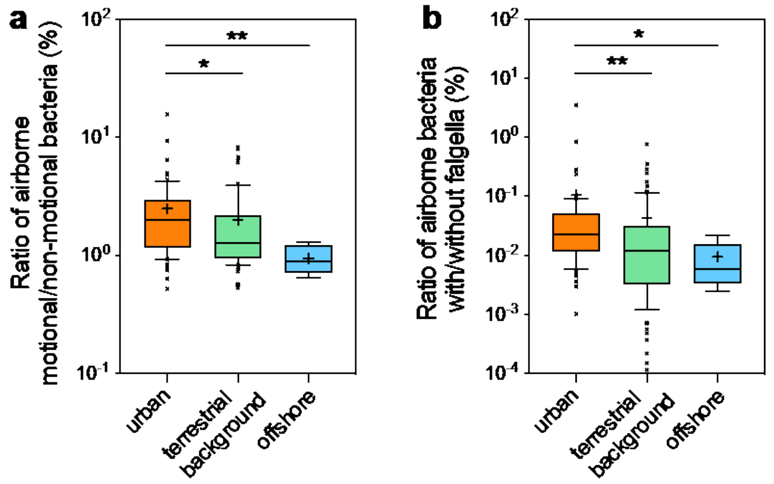

Fig. S17. Motility of global airborne bacteria in urban, terrestrial background, and offshore areas. (a) Ratio of airborne motional to non-motional bacteria. (b) Ratio of airborne bacteria with flagella to bacteria without flagella.

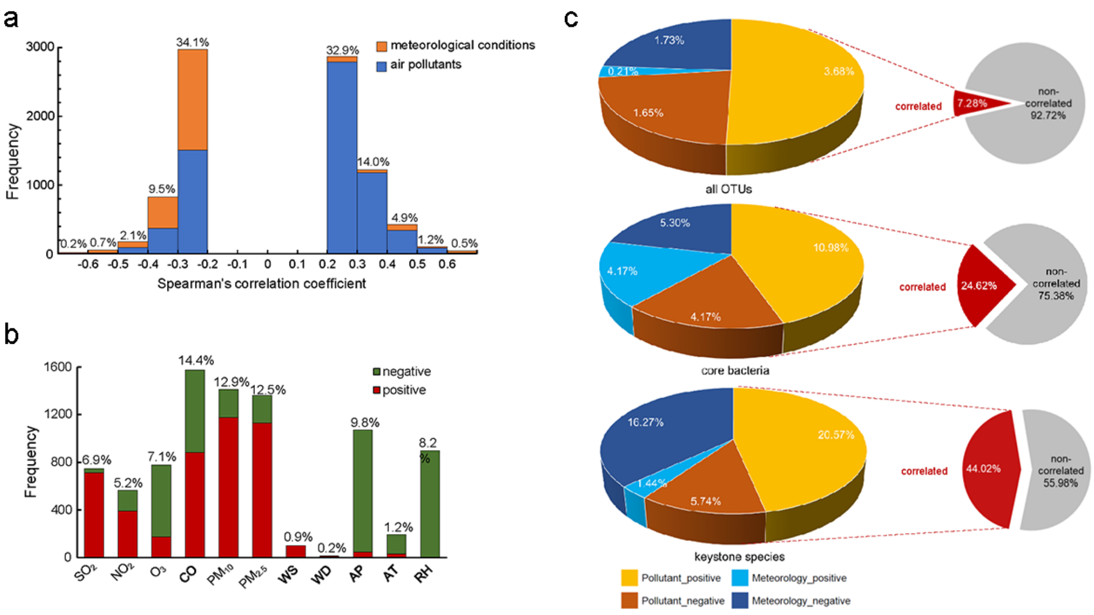

Fig. S18. Correlation distribution between airborne bacterial community composition and environmental factors (air pollutants and meteorological conditions). (*a*) Histogram of the correlation frequency showing the distribution of Spearman's correlation coefficients (R-value) between whole global airborne bacterial communities (10,897 OTUs) and environmental factors. Only significant correlations ($p < 0.05$) were counted in the histogram. The percentage number above each pillar indicates the proportion of significant relationships that fall within that section. (*b*) Histogram of the correlation frequency showing the distribution of significant correlations ($p < 0.05$) with diverse environmental factors in whole global airborne bacterial communities (10,897 OTUs). The percentage number above each pillar indicates the probability of the occurrence of a significant correlation of that factor with OTUs. (*c*) Pie charts of correlation frequencies showing the probability of significant relationships (positive or negative, affected by air pollutants or meteorological conditions) occurring in whole airborne bacterial communities, keystone species communities, and core bacterial communities. The right panel highlights the ratio of the number of existing significant relationships ($p < 0.05$) to the number of all possible connections between OTUs and environmental variables. The left panel classifies all of these existing significant correlations into four groups and gives an indication of the strength of the correlation through a percentage value.

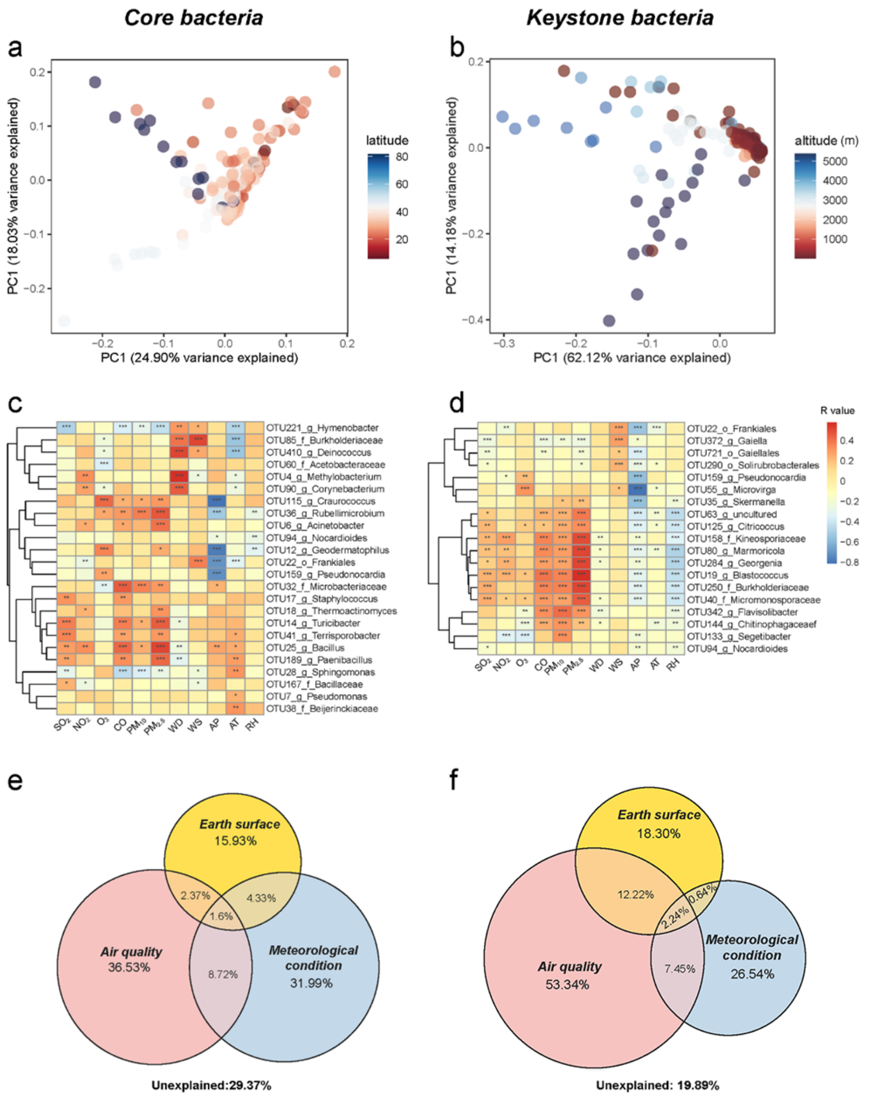

Fig. S19. Impacts of environmental factors on core and keystone bacterial communities in the global atmosphere. (*a* and *b*) PCoA performed on dissimilarities in the composition of global airborne core and keystone bacterial communities on the basis of a "Euclidean" index of 370 air samples. Samples are color coded according to the corresponding geographic locations. (*c* and *d*) The correlations of 24 core bacteria and 19 keystone species with environmental variables. The right color gradient indicates Spearman's rank correlation coefficients (i.*e.,* R), with dark red indicating stronger positive correlations and dark blue indicating stronger negative correlations. The number of asterisks indicates the significance levels (two-sided) of the Spearman's rank correlation coefficients (*** $p < 0.001$, ** $p < 0.01$, * $p < 0.05$). Each row represents the correlation of a specific OTU and environmental variables in 370 biologically independent air samples, which are clustered based on Spearman's rank correlation coefficients. (*e* and *f*) VPA showing relative contributions of air quality (PM_{10}, $PM_{2.5}$, SO_2, NO_2, O_3, and CO), meteorological condition (air temperature, air pressure, relative humidity, wind speed, and wind direction), and landscape coverage type (water/sea, urban, grassland, cropland, forest, and shrubs) to community variations in core bacteria and keystone species. The overlap represents the joint effect explained by two or three factor groups together, while the percentage number below each group name represents the variance explained by one group alone. "Unexplained" denotes a variance that could not be explained by any one of these three groups.

Table S1. The taxonomic composition of 24 global airborne core bacteria (phylum, class, order, family, and genus level).

OTU number	Mean abundance (0.01%)	Phylum	Class	Order	Family	Genus
OTU4	204.75	Proteobacteria	Alphaproteobacteria	Rhizobiales	Beijerinckiaceae	Methylobacterium
OTU28	153.25	Proteobacteria	Alphaproteobacteria	Sphingomonadales	Sphingomonadaceae	Sphingomonas
OTU25	150.75	Firmicutes	Bacilli	Bacillales	Bacillaceae	Bacillus
OTU221	142.00	Bacteroidetes	Bacteroidia	Cytophagales	Hymenobacteraceae	Hymenobacter
OTU14	114.00	Firmicutes	Erysipelotrichia	Erysipelotrichales	Erysipelotrichaceae	Turicibacter
OTU18	107.98	Firmicutes	Bacilli	Bacillales	Thermoactinomycetaceae	Thermoactinomyces
OTU6	96.16	Proteobacteria	Gammaproteobacteria	Pseudomonadales	Moraxellaceae	Acinetobacter
OTU22	87.82	Actinobacteria	Actinobacteria	Frankiales	uncultured	uncultured_ge
OTU7	77.39	Proteobacteria	Gammaproteobacteria	Pseudomonadales	Pseudomonadaceae	Pseudomonas
OTU36	74.88	Proteobacteria	Alphaproteobacteria	Rhodobacterales	Rhodobacteraceae	Rubellimicrobium
OTU167	63.12	Firmicutes	Bacilli	Bacillales	Bacillaceae	Bacillaceae_unclassified
OTU12	60.82	Actinobacteria	Actinobacteria	Frankiales	Geodermatophilaceae	Geodermatophilus
OTU41	54.44	Firmicutes	Clostridia	Clostridiales	Peptostreptococcaceae	Terrisporobacter
OTU32	53.83	Actinobacteria	Actinobacteria	Micrococcales	Microbacteriaceae	Microbacteriaceae_unclassified
OTU17	50.39	Firmicutes	Bacilli	Bacillales	Staphylococcaceae	Staphylococcus
OTU38	48.42	Proteobacteria	Alphaproteobacteria	Rhizobiales	Beijerinckiaceae	1174-901-12
OTU410	48.07	Deinococcus-Thermus	Deinococci	Deinococcales	Deinococcaceae	Deinococcus
OTU94	47.88	Actinobacteria	Actinobacteria	Propionibacteriales	Nocardioidaceae	Nocardioides
OTU115	46.71	Proteobacteria	Alphaproteobacteria	Acetobacterales	Acetobacteraceae	Craurococcus
OTU85	45.67	Proteobacteria	Gammaproteobacteria	Betaproteobacteriales	Burkholderiaceae	Burkholderiaceae_unclassified
OTU60	32.34	Proteobacteria	Alphaproteobacteria	Acetobacterales	Acetobacteraceae	Acetobacteraceae_unclassified
OTU189	31.95	Firmicutes	Bacilli	Bacillales	Paenibacillaceae	Paenibacillus
OTU90	30.92	Actinobacteria	Actinobacteria	Corynebacteriales	Corynebacteriaceae	Corynebacterium_1
OTU159	30.20	Actinobacteria	Actinobacteria	Pseudonocardiales	Pseudonocardiaceae	Pseudonocardia

Table S2. Taxonomic information and relative abundance of core communities in air, marine, and soil habitats based on the global datasets (22, 65).

Global air		Global marine		Global soil	
taxonomy	mean abundance (0.01%)	taxonomy	mean abundance (0.01%)	taxonomy	mean abundance (0.01%)
g_Methylobacterium	204.75	f_Surface 1	1784.32	g_Subgroup_6_ge	417.06
g_Sphingomonas	153.25	f_SAR86 clade	853.58	g_Candidatus_Udaeobacter	339.68
g_Bacillus	150.75	o_SAR11 clade	789.24	f_Xanthobacteraceae	215.98
g_Hymenobacter	142.00	g_Prochlorococcus	747.39	g_Acidothermus	191.05
g_Turicibacter	114.00	g_AEGEAN-169 marine group	331.23	f_Gemmataceae	175.25
g_Thermoactinomyces	107.98	f_Rhodobacteraceae	318.25	f_Xanthobacteraceae	164.15
g_Acinetobacter	96.16	f_SAR116 clade	283.27	g_RB41	156.39
o_Frankiales	87.82	f_OCS155 marine group	269.20	g_67-14_ge	155.74
g_Pseudomonas	77.39	f_SAR406 clade (Marine group A)	266.45	g_uncultured_ge	137.16
g_Rubellimicrobium	74.88	f_Marine Group II	228.16	f_Xanthobacteraceae	127.60
f_Bacillaceae	63.12	g_Candidatus Pelagibacter	225.97	o_Acidobacteriales	118.46
g_Geodermatophilus	60.82	f_Surface 2	212.47	g_WD2101_soil_group_ge	115.17
g_Terrisporobacter	54.44	g_NS5 marine group	130.67	g_Acidibacter	114.52
f_Microbacteriaceae	53.83	g_NS4 marine group	115.99	g_KD4-96_ge	111.71
g_Staphylococcus	50.39	g_Marinicella	80.53	o_Gaiellales	110.52
g_1174-901-12	48.42	f_Surface 4	73.32	g_Mycobacterium	100.56
g_Deinococcus	48.07			o_Gaiellales	99.99
g_Nocardioides	47.88			g_Candidatus_Solibacter	80.32
g_Craurococcus	46.71			g_Bryobacter	80.02
f_Burkholderiaceae	45.67			g_Reyranella	70.93
f_Acetobacteraceae	32.34			c_Alphaproteobacteria	68.74
g_Paenibacillus	31.95			g_Haliangium	67.63
g_Corynebacterium_1	30.92			g_Solirubrobacter	65.08
g_Pseudonocardia	30.20			g_IMCC26256_ge	61.50
				g_Chthoniobacter	61.36
				g_TK10_ge	51.65
				g_MND1	49.24

Table S3. The topological properties of the co-occurrence network.

Topological property	Definition & Function	Air	Soil	Marine
number of nodes	nodes: interconnected objects (bacteria)	482	490	482
number of edges	edges: links between each pair of nodes	5,038	3,323	7,629
number of clusters	clusters: groups of objects with tight interactions	3	1	2
average shortest path length	average network distance between all pairs of nodes	5.24	3.03	3.97
diameter	maximum of pairwise distances between every two nodes	15	9	10
clustering coefficient	the degree to which nodes tend to cluster together	0.67	0.48	0.58

Table S4. The taxonomic composition of 19 global airborne keystone bacteria (phylum, class, order, family, and genus level).

OTU number	Mean abundance (0.01%)	Phylum	Class	Order	Family	Genus
OTU94	47.88	Actinobacteria	Actinobacteria	Propionibacteriales	Nocardioidaceae	Nocardioides
OTU22	87.82	Actinobacteria	Actinobacteria	Frankiales	uncultured	uncultured_ge
OTU40	8.27	Actinobacteria	Actinobacteria	Micromonosporales	Micromonosporaceae	Micromonosporaceae_unclassified
OTU125	1.70	Actinobacteria	Actinobacteria	Micrococcales	Micrococcaceae	Citricoccus
OTU80	3.87	Actinobacteria	Actinobacteria	Propionibacteriales	Nocardioidaceae	Marmoricola
OTU158	2.51	Actinobacteria	Actinobacteria	Kineosporiales	Kineosporiaceae	Kineosporiaceae_unclassified
OTU19	22.16	Actinobacteria	Actinobacteria	Frankiales	Geodermatophilaceae	Blastococcus
OTU284	1.01	Actinobacteria	Actinobacteria	Micrococcales	Bogoriellaceae	Georgenia
OTU159	30.20	Actinobacteria	Actinobacteria	Pseudonocardiales	Pseudonocardiaceae	Pseudonocardia
OTU372	12.27	Actinobacteria	Thermoleophilia	Gaiellales	Gaiellaceae	Gaiella
OTU721	13.63	Actinobacteria	Thermoleophilia	Gaiellales	uncultured	uncultured_ge
OTU290	26.05	Actinobacteria	Thermoleophilia	Solirubrobacterales	67-14	67-14_ge
OTU133	51.66	Bacteroidetes	Bacteroidia	Chitinophagales	Chitinophagaceae	Segetibacter
OTU342	5.87	Bacteroidetes	Bacteroidia	Chitinophagales	Chitinophagaceae	Flavisolibacter
OTU144	4.04	Bacteroidetes	Bacteroidia	Chitinophagales	Chitinophagaceae	Chitinophagaceae_unclassified
OTU55	30.65	Proteobacteria	Alphaproteobacteria	Rhizobiales	Beijerinckiaceae	Microvirga
OTU63	3.71	Proteobacteria	Alphaproteobacteria	Rhizobiales	Beijerinckiaceae	uncultured
OTU35	35.30	Proteobacteria	Alphaproteobacteria	Azospirillales	Azospirillaceae	Skermanella
OTU250	1.15	Proteobacteria	Gammaproteobacteria	Betaproteobacteriales	Burkholderiaceae	Burkholderiaceae_unclassified

Table S5. Summary of keystone taxa reported in the literature on different ecosystems and habitats.

Ecosystem or habitat		Keystone taxa	Reference
Aquatic ecosystems	shallow lake	Cyanobacteria	(66)
	freshwater sediment	Planctomycetes, Proteobacteria, Nitrospira, Clostridia, Actinobacteria, Bacilli	(67)
	nearshore	Verrucomicrobiaceae, Crenarchaeaceae, Candidatus OP3, Chloroflexi,Chloracidobacteria	(68)
	seawater_culture	Rhodobacteraceae, Alteromonadaceae	(69)
Forests	Brazilian forest	Burkholderiales, Acidimicrobiaceae, Rhodospirillaceae	(70)
	Chinese forest	Rubrobacteriales, Gaiellales, Rhizobiales, Gaiellales, Solirubrobacteriales	(71)
Agricultural lands	soybean soil	Rhizobiales, Methylophilaceae, Acetobacteraceae	(70)
	sugarcane soil	Burkholderiales, Caulobacter, Kineosporia, Sporichthya	(70)
	blueberry soil	Frankiales, Pseudomonas, Burkholderia	(72)
	paddy soil	Rhodobiaceae, Hydrogenophilaceae, Comamonadaceae, Alcaligenaceae, Nitrosomonadaceae	(73)
	Pinus plantation	Sporichthya, Burkholderiales	(70)
Arctic & Antarctic ecosystems	Antarctic lake	Acidobacteriaceae	(74)
	Arctic soil	Burkholderia, Bradyrhizobium, Rhodoplanes, Pseudomonas	(75)
	permafrost	Burkholderiales, Actinomycetales, Rhizobiales, Puniceicoccaceae	(76)
	permafrost soils	Burkholderia, Bradyrhizobium, Phenylobacterium	(77)
	Arctic ice cap	Actinobacteria	(77)

Table S6. Sampling frequency and number of air subsamples in this study.

Region	Type	Sampling Period	Sampling Frequency	Sample size
Beijing (northern China)	Urban-1	Apr 2016 - May 2017	weekly	61
	Urban-2	Dec 2016 - Nov 2017	weekly	44
	Rural	Aug 2018- May 2019	weekly	33
Nanjing (eastern China)	Industrial	Mar 2016- May 2017	Every 7-10 days	46
	Urban	Mar 2016 - May 2017	Every 7-10 days	48
	Rural	Apr 2016 - May 2017	Around every month	18
Guangzhou (southern China)	Urban	Mar 2016 - May 2017	Around every 3 days	122
	Suburban	Mar 2016 - May 2017	Around weekly	52
	Semi-rural	Mar 2016 - May 2017	weekly	65
Hong Kong	Urban	Jan 2016 – Nov 2016	Around every month	16
Phitsanulok, Thailand	Urban	Jan 2016 – Dec 2016	Around weekly	37
Bachok, Malaysia	Urban	Dec 2015 – Nov 2016	Around weekly	40
Tibet	Pristine	Nov 2017 – Feb 2019	Daily or weekly	122
Waliguan	Background	Oct 2014 – Jun 2015	Around 4 days	22
Changbai Mountain	Background	Sep 2013 – Jul 2014	Around 2-3 days	23
Ailao Mountain	Background	Apr 2014 – May 2015	Around weekly	36
Hok Tsui	Background	Nov 2013 – Aug 2014	Around weekly	17

Table S7. The overview of climate types and land cover types of 370 air samples in 63 sampling sites.

Sites	Climate system	Sample size	Sample ID in Table S2	Land coverage type composition (%)					
				Water/Seas	Forest	Shrubs	Grassland	Cropland	Built-up
Bachok, Malaysia	Af	4	No.17 - 20	0.00%	0.00%	3.21%	10.29%	84.88%	1.62%
Phitsanulok, Thailand	Aw	4	No.21 - 24	54.27%	5.69%	29.12%	7.02%	3.52%	0.38%
Tsogt-Ovoo, Mongolia	BWk	12	No.279 - 290	0.00%	0.00%	59.78%	0.00%	0.00%	40.22%
Nanjing_ rural, China	Cfa	5	No.40 - 44	19.08%	0.09%	24.14%	17.68%	39.00%	0.00%
Nanjing_ urban, China	Cfa	5	No.45 - 49	10.97%	0.61%	19.39%	21.79%	43.22%	4.02%
Nanjing_ industrial, China	Cfa	5	No.50 - 54	3.91%	0.60%	19.01%	29.72%	46.23%	0.53%
Ohio 1, USA	Cfa	6	No.308 - 313	34.85%	0.95%	33.04%	1.02%	10.25%	19.88%
Ohio 2, USA	Cfa	6	No.314 - 319	1.45%	1.77%	12.02%	10.71%	11.70%	62.34%
Ljungbyhed, Sweden	Cfb	46	No.168 - 213	1.50%	39.00%	26.79%	1.23%	31.39%	0.10%
Wiesbaden, Germany	Cfb	43	No.328 - 370	2.12%	18.37%	16.49%	4.29%	54.26%	4.48%
Cape Point, South Africa	Csb	7	No.86 -92	93.55%	0.04%	5.74%	0.18%	0.03%	0.47%
Hok Tsui, China	Cwa	4	No.5 - 8	89.82%	0.86%	6.10%	2.16%	0.00%	1.07%
Mt. Ailao, China	Cwa	4	No.9 - 12	0.00%	83.19%	11.61%	1.15%	4.05%	0.00%
Guangzhou_ urban, China	Cwa	5	No.25-29	7.44%	0.02%	14.11%	44.69%	30.63%	3.11%
Guangzhou_ suburban, China	Cwa	5	No.30 - 34	9.37%	0.00%	22.47%	26.89%	40.86%	0.41%
Guangzhou_ semi-rural, China	Cwa	5	No.35 - 39	2.03%	15.09%	36.29%	5.96%	40.62%	0.00%
Hong Kong, China	Cwa	4	No.65 - 68	67.01%	2.31%	8.08%	12.81%	8.59%	1.19%
Chacaltaya, Bolivia	Cwb	16	No.93 - 108	0.00%	2.05%	52.86%	40.97%	4.11%	0.00%
STP, USA	Dfa	4	No.164 - 167	0.12%	0.00%	39.92%	10.82%	1.83%	47.32%
Colorado 1, USA	Dfa	2	No.291 - 292	0.26%	0.00%	9.95%	46.81%	31.78%	11.21%
Colorado 2, USA	Dfa	1	No.293	0.00%	65.36%	20.40%	11.39%	2.52%	0.33%
Colorado 3, USA	Dfa	1	No.294	0.23%	0.00%	8.22%	37.82%	49.67%	4.06%

Sites	Climate system	Sample size	Sample ID in Table S2	Land coverage type composition (%)					
				Water/Seas	Forest	Shrubs	Grassland	Cropland	Built-up
Colorado 4, USA	Dfa	1	No.295	0.23%	0.97%	16.92%	33.48%	45.18%	3.23%
Colorado 5, USA	Dfa	1	No.296	0.00%	0.00%	49.63%	29.55%	16.82%	4.00%
Colorado 6, USA	Dfa	1	No.297	0.00%	36.76%	51.88%	9.77%	1.59%	0.00%
Colorado 7, USA	Dfa	1	No.298	0.00%	6.74%	14.76%	32.33%	43.99%	2.18%
Colorado 8, USA	Dfa	8	No.299 - 306	0.00%	60.62%	24.01%	3.29%	12.09%	0.00%
Colorado 9, USA	Dfa	1	No.307	1.18%	6.33%	16.04%	43.38%	31.09%	1.99%
Michigan 1, USA	Dfa	5	No.320-324	12.49%	0.00%	2.82%	2.58%	23.51%	58.59%
Michigan 2, USA	Dfa	3	No.325 - 327	0.73%	1.13%	51.31%	0.78%	45.53%	0.52%
Pic-du-Midi, France	Dfb	12	No.139 - 150	0.00%	24.29%	10.52%	0.00%	65.01%	0.18%
Kiruna, Sweden	Dfb	46	No.214 - 259	9.45%	3.62%	84.53%	1.38%	0.00%	1.02%
Grenoble, France	Dfc	10	No.109 - 118	0.67%	61.40%	26.86%	3.08%	7.48%	0.51%
PuydeDôme, France	Dfc	12	No.127 - 138	0.70%	31.72%	46.33%	2.55%	18.32%	0.38%
Beijing_ urban 1, China	Dwa	5	No.55 -59	0.25%	0.14%	5.80%	48.67%	30.80%	14.33%
Beijing_ urban 2, China	Dwa	5	No.60 -64	0.25%	0.14%	5.80%	48.67%	30.80%	14.33%
Beijing_ rural, China	Dwa	4	No.73 - 76	4.16%	6.09%	60.54%	3.85%	25.36%	0.00%
Mt. Changbai, China	Dwb	4	No.1 - 4	0.11%	36.04%	55.91%	0.91%	6.98%	0.05%
Waliguan, China	Dwc	4	No.13 - 16	0.00%	0.00%	44.85%	34.66%	19.24%	1.24%
Mt. Everest	ET	4	No.69 - 72	0.00%	0.00%	46.38%	2.92%	0.00%	50.71%
Namco, China	ET	8	No.119 - 126	22.63%	0.00%	14.45%	62.74%	0.00%	0.18%
Station-Nord, Greenland	ET	13	No.151 - 163	100.00%	0.00%	0.00%	0.00%	0.00%	0.00%
Amsterdam-Island, France	Offshore	9	No.77 - 85	100.00%	0.00%	0.00%	0.00%	0.00%	0.00%
South Ocean (19 sites)	Offshore	19	No.260 - 278	100.00%	0.00%	0.00%	0.00%	0.00%	0.00%

Notes:

4 samples in tropical rainforest climates (*Af*), 4 samples in tropical savanna climates (*Aw*), 12 samples in cold desert climates (*BWk*), 27 samples in dry-winter humid subtropical climates (*Cwa*), 16 samples in dry-winter subtropical highland climates (*Cwb*), 27 samples in humid subtropical climates (*Cfa*), 89 samples in temperate oceanic climates (*Cfb*), 7 samples in Mediterranean warm/cool summer climates (*Csb*), 29 samples in hot-summer humid continental climates (*Dfa*), 58 samples in warm-summer humid continental climates (*Dfb*), 22 samples in subarctic climates (*Dfc*), 14 samples in monsoon-influenced hot-summer humid continental climates (*Dwa*), 4 samples in monsoon-influenced warm-summer humid continental climates (*Dwb*), 4 samples in monsoon-influenced subarctic climates (*Dwc*), 25 samples in tundra climates (*ET*), and 28 samples collected from offshore areas.

Additional Dataset 1 (SI – B, a separate file)

Basic sampling information, sequence number, and bacterial richness of samples used in this study.

Supplementary references

1. M. A. Friedl, Global land cover mapping from MODIS: algorithms and early results. *Remote Sens. Environ.* **83,** 287-302 (2002).
2. S. M. Burrows, T. Butler, P. Jockel, H. Tost, A. Kerkweg, Bacteria in the global atmosphere – Part 2: Modeling of emissions and transport between different ecosystems. *Atmos. Chem. Phys.* **9,** 9281-9297 (2009).
3. S. Chen, Y. Zhou, Y. Chen, J. Gu, fastp: an ultra-fast all-in-one FASTQ preprocessor. *Bioinformatics* **34,** i884-i890 (2018).
4. D. E. Wood, J. Lu, B. Langmead, Improved metagenomic analysis with Kraken 2. *Genome Biol.* **20,** 257 (2019).
5. J. Lu, F. P. Breitwieser, P. Thielen, S. L. Salzberg, Bracken: estimating species abundance in metagenomics data. *Peer J. Comput. Sci.* **3,** e104 (2017).
6. D. Liu et al., The use of levoglucosan and radiocarbon for source apportionment of PM(2.5) carbonaceous aerosols at a background site in East China. *Environ. Sci. Technol.* **47,** 10454-10461 (2013).
7. A. M. Saunders, M. Albertsen, J. Vollertsen, P. H. Nielsen, The activated sludge ecosystem contains a core community of abundant organisms. *ISME. J.* **10,** 11-20 (2016).
8. A. J. Szekely, S. Langenheder, The importance of species sorting differs between habitat generalists and specialists in bacterial communities. *FEMS Microbiol. Ecol.* **87,** 102-112 (2014).
9. S. W. Kembel et al., Picante: R tools for integrating phylogenies and ecology. *Bioinformatics* **26,** 1463-1464 (2010).
10. F. Jari Oksanen et al., vegan: Community Ecology Package. R package, Version 2.0-4. Available at http://CRAN.R-project.org/package=vegan. (2018).
11. R. J. Hijmans, E. Williams, C. Vennes, geosphere: Spherical Trigonometry. R package, Version 1.5-10. Available at https://cran.r-project.org/package=geosphere. (2015).
12. P. Langfelder, S. Horvath, Fast R Functions for robust correlations and hierarchical clustering. *J. Stat. Softw.* **46,** i11(2012).
13. Anonymous, Correlation Networks. In Analysis of Biological Networks. Y. Pan, A.Y. Zomaya, B.H. Junker, F. Schreiber, Eds. (2008).
14. Gabor Csardi et al., igraph: Network Analysis and Visualization. R package, Version 1.2.5. Available at https://igraph.org/. (2020).
15. D. Berry, S. Widder, Deciphering microbial interactions and detecting keystone species with co-occurrence networks. *Front Microbiol.* **5,** 219 (2014).
16. S. Banerjee, K. Schlaeppi, M. G. A. van der Heijden, Keystone taxa as drivers of microbiome structure and functioning. *Nat. Rev. Microbiol.* **16,** 567-576 (2018).
17. M. D. Humphries, K. Gurney, Network 'small-world-ness': a quantitative method for determining canonical network equivalence. *PLoS One* **3,** e0002051 (2008).
18. J. W. Duncan, H. S. Steven, Collective dynamics of 'small-world' networks. *Nature* **393,** 3 (1998).
19. K. J. Locey, J. T. Lennon, Scaling laws predict global microbial diversity. *Proc. Natl. Acad. Sci. U.S.A.* **113,** 5970-5975 (2016).

20. D. J. Smith, Microbes in the upper atmosphere and unique opportunities for astrobiology research. *Astrobiology* **13,** 981-990 (2013).
21. G. Horneck, D. M. Klaus, R. L. Mancinelli, Space microbiology. *Microbiol. Mol. Biol. Rev.* **74,** 121-156 (2010).
22. M. Bahram et al., Structure and function of the global topsoil microbiome. *Nature* **560,** 233-237 (2018).
23. W. B. Whitman, D. C. Coleman, W. J. Wiebe, Prokaryotes: The unseen majority. *Proc. Natl. Acad. Sci. U.S.A.* **95,** 6278-6583 (1998).
24. J. Kallmeyer, R. Pockalny, R. R. Adhikari, D. C. Smith, S. D'Hondt, Global distribution of microbial abundance and biomass in subseafloor sediment. *Proc. Natl. Acad. Sci. U.S.A.* **109,** 16213-16216 (2012).
25. L. R. Thompson et al., A communal catalogue reveals Earth's multiscale microbial diversity. *Nature* **551,** 457-463 (2017).
26. E. Bolyen et al., Reproducible, interactive, scalable and extensible microbiome data science using QIIME 2. *Nat. Biotechnol.* **37,** 852-857 (2019).
27. D. Knights et al., Bayesian community-wide culture-independent microbial source tracking. *Nat. Methods* **8,** 761-763 (2011).
28. D. Ning et al., A quantitative framework reveals ecological drivers of grassland microbial community assembly in response to warming. *Nat. Commun.* **11,** 4717 (2020).
29. Y. Rosseel, lavaan: an R package for structural equation modeling. *J. Stat. Softw.* **48,** 36 (2012).
30. Z. Zhang, J. Zhang, A big world inside small-world networks. *PLoS One* **4,** e5686 (2009).
31. M. E. J. Newman, The structure and function of complex networks*. *SIAM Review* **45,** 167-256 (2003).
32. A. L. Barabasi, Scale-Free Networks: A decade and beyond. *Science* **325,** 412-413 (2009).
33. A. Parte et al., "The Actinobacteria" in *Bergey's Manual of Systematic Bacteriology*, W. B. Whitman, Ed. (Springer Science+Business Media, 2012), vol. 5, pp. 509.
34. R. Paine, Food web complexity and species diversity. *Am. Nat.* **100,** 65-75 (1966).
35. F. D. Andreote et al., Culture-independent assessment of Rhizobiales-related alphaproteobacteria and the diversity of Methylobacterium in the rhizosphere and rhizoplane of transgenic eucalyptus. *Microb. Ecol.* **57,** 82-93 (2009).
36. L. Nazaries, J. C. Murrell, P. Millard, L. Baggs, B. K. Singh, Methane, microbes and models: fundamental understanding of the soil methane cycle for future predictions. *Environ. Microbiol.* **15,** 2395-2417 (2013).
37. L. Albuquerque, M. S. Costa, "The family Gaiellaceae" in *The Prokaryotes: Actinobacteria,* E. Rosenberg, E. F. DeLong, S. Lory, E. Stackebrandt, F. Thompson, Eds. (Springer Berlin Heidelberg, Berlin, Heidelberg, 2014), pp. 357-360.
38. H. E. W. Cottee-Jones, R. J. Whittaker, The keystone species concept: a critical appraisal. *Frontiers of Biogeography* **4,** 217-220 (2012).
39. R. A. Stern et al., The microbiome of size-fractionated airborne particles from the Sahara region. *Environ. Sci. Technol.* **55,** 1487-1496 (2021).

40. R. Lu et al., Bacterial community structure in atmospheric particulate matters of different sizes during the haze days in Xi'an, China. *Sci. Total. Environ.* **637-638,** 244-252 (2018).
41. D. Yan et al., Structural Variation in the Bacterial Community Associated with Airborne Particulate Matter in Beijing, China, during Hazy and Nonhazy Days. *Appl. Environ. Microbiol.* **84,** e00004-00018 (2018).
42. H. Gou, C. Xie, Y. Tong, S. Li, J. Lu, Assessment of microbial communities in TSP and PM_{10} of Shihezi during spring. *Chinese J. Environ. Eng.* **11,** 2343-2349 (2017).
43. R. M. Harrison et al., Climate factors influencing bacterial count in background air samples. *Int. J. Biometeorol* **49,** 167-178 (2005).
44. R. M. Bowers et al., Characterization of airborne microbial communities at a high-elevation site and their potential to act as atmospheric ice nuclei. *Appl. Environ. Microbiol.* **75,** 5121-5130 (2009).
45. S. H. Lee et al., Identification of airborne bacterial and fungal community structures in an urban area by T-RFLP analysis and quantitative real-time PCR. *Sci. Total. Environ.* **408,** 1349-1357 (2010).
46. B. C. Cho, C. Y. Hwang, Prokaryotic abundance and 16S rRNA gene sequences detected in marine aerosols on the East Sea (Korea). *FEMS Microbiol. Ecol.* **76,** 327-341 (2011).
47. V. Bertolini et al., Temporal variability and effect of environmental variables on airborne bacterial communities in an urban area of Northern Italy. *Appl. Microbiol. Biotechnol* **97,** 6561-6570 (2013).
48. R. M. Bowers, I. B. McCubbin, A. G. Hallar, N. Fierer, Seasonal variability in airborne bacterial communities at a high-elevation site. *Atmos. Environ.* **50,** 41-49 (2012).
49. N. DeLeon-Rodriguez et al., Microbiome of the upper troposphere: species composition and prevalence, effects of tropical storms, and atmospheric implications. *Proc. Natl. Acad. Sci. U.S.A.***110,** 2575-2580 (2013).
50. K. Murata, D. Zhang, Transport of bacterial cells toward the Pacific in Northern Hemisphere westerly winds. *Atmos. Environ*. **87,** 138-145 (2014).
51. D. Tanaka, Y. Terada, T. Nakashima, A. Sakatoku, S. Nakamura, Seasonal variations in airborne bacterial community structures at a suburban site of central Japan over a 1-year time period using PCR-DGGE method. *Aerobiologia* **31,** 143-157 (2014).
52. N. Yamaguchi, T. Ichijo, T. Baba, M. Nasu, Long-range transportation of bacterial cells by Asian dust. *Genes Environ.* **36,** 145-151 (2014).
53. A. Barberan et al., Continental-scale distributions of dust-associated bacteria and fungi. *Proc. Natl. Acad. Sci. U.S.A.* **112,** 5756-5761 (2015).
54. W. Deng et al., Distribution of bacteria in inhalable particles and its implications for health risks in kindergarten children in Hong Kong. *Atmos. Environ.* **128,** 268-275 (2016).
55. J.-F. Gao, X.-Y. Fan, H.-Y. Li, K.-L. Pan, Airborne bacterial communities of $PM_{2.5}$ in Beijing-Tianjin-Hebei megalopolis, China as revealed by Illumina MiSeq sequencing: A case study. *Aerosol Air Qual. Res.* **17,** 788-798 (2017).
56. D. Gat, Y. Mazar, E. Cytryn, Y. Rudich, Origin-dependent variations in the atmospheric microbiome community in eastern Mediterranean dust storms. *Environ. Sci. Technol.* **51,** 6709-6718 (2017).

57. S. Genitsaris et al., Variability of airborne bacteria in an urban Mediterranean area (Thessaloniki, Greece). *Atmos. Environ.* **157,** 101-110 (2017).
58. E. Innocente et al., Influence of seasonality, air mass origin and particulate matter chemical composition on airborne bacterial community structure in the Po Valley, Italy. *Sci. Total. Environ.* **593-594,** 677-687 (2017).
59. C. Xu et al., Bacterial characterization in ambient submicron particles during severe haze episodes at Ji'nan, China. *Sci. Total. Environ.* **580,** 188-196 (2017).
60. Q. Zhen et al., Meteorological factors had more impact on airborne bacterial communities than air pollutants. *Sci. Total. Environ.* **601-602,** 703-712 (2017).
61. T. Santl-Temkiv, U. Gosewinkel, P. Starnawski, M. Lever, K. Finster, Aeolian dispersal of bacteria in southwest Greenland: their sources, abundance, diversity and physiological states. *FEMS Microbiol. Ecol.* **94** (2018).
62. F. Shen, M. Niu, F. Zhou, Y. Wu, T. Zhu, Culturability, metabolic activity and composition of ambient bacterial aerosols in a surrogate lung fluid. *Sci. Total. Environ.* **690,** 76-84 (2019).
63. R. Tignat-Perrier et al., Global airborne microbial communities controlled by surrounding landscapes and wind conditions. *Sci. Rep.* **9,** 14441 (2019).
64. C. Xu et al., Profile of inhalable bacteria in PM2.5 at Mt. Tai, China: Abundance, community, and influence of air mass trajectories. *Ecotoxicol Environ. Saf.* **168,** 110-119 (2019).
65. S. Sunagawa et al., Structure and function of the global ocean microbiome. *Science* **348,** 1261359 (2015).
66. D. Zhao et al., Network analysis reveals seasonal variation of co-occurrence correlations between Cyanobacteria and other bacterioplankton. *Sci. Total. Environ.* **573,** 817-825 (2016).
67. Y. Ji et al., Structure and function of methanogenic microbial communities in sediments of Amazonian lakes with different water types. *Environ. Microbiol.* **18,** 5082-5100 (2016).
68. E. B. Graham et al., Deterministic influences exceed dispersal effects on hydrologically-connected microbiomes. *Environ. Microbiol.* **19,** 1552-1567 (2017).
69. H. Geng, M. B. Tran-Gyamfi, T. W. Lane, K. L. Sale, E. T. Yu, Changes in the structure of the microbial community associated with Nannochloropsis salina following treatments with antibiotics and bioactive compounds. *Front Microbiol.* **7,** 1155 (2016).
70. M. Lupatini et al., Network topology reveals high connectance levels and few key microbial genera within soils. *Front. Environ. Sci.* **2,** 1–11 (2014).
71. B. Ma et al., Geographic patterns of co-occurrence network topological features for soil microbiota at continental scale in eastern China. *ISME. J.* **10,** 1891-1901 (2016).
72. Y. Jiang et al., Plant cultivars imprint the rhizosphere bacterial community composition and association networks. *Soil Biol. Biochem.* **109,** 145-155 (2017).
73. H. Wang et al., Combined use of network inference tools identifies ecologically meaningful bacterial associations in a paddy soil. *Soil Biol. Biochem.* **105,** 227-235 (2017).
74. T. J. Vick-Majors, J. C. Priscu, L. A. Amaral-Zettler, Modular community structure suggests metabolic plasticity during the transition to polar night in ice-covered Antarctic lakes. *ISME. J.* **8,** 778-789 (2014).
75. R. Hill et al., Temporal and spatial influences incur reconfiguration of Arctic heathland soil bacterial community structure. *Environ. Microbiol.* **18,** 1942-1953 (2016).

76. J. Comte, C. Lovejoy, S. Crevecoeur, W. F. Vincent, Co-occurrence patterns in aquatic bacterial communities across changing permafrost landscapes. *Biogeosciences* **13,** 175-190 (2016).
77. J. K. Gokul et al., Taxon interactions control the distributions of cryoconite bacteria colonizing a High Arctic ice cap. *Mol. Ecol.* **25,** 3752-3767 (2016).
